# Assessing image quality in photoacoustic imaging: A metric-based and deep learning-based evaluation

**DOI:** 10.1016/j.pacs.2026.100800

**Published:** 2026-02-28

**Authors:** Melle Van Der Brugge, Kalloor Joseph Francis, Navchetan Awasthi

**Affiliations:** a Faculty of Science, Mathematics and Computer Science, Informatics Institute, University of Amsterdam, Amsterdam, 1090 GH, The Netherlands; b Erasmus MC, Cardiovascular Institute, Department of Cardiology, Biomedical Engineering, Rotterdam, The Netherlands; c School of Artificial Intelligence and Data Science, IIT Jodhpur, Rajasthan, 342030, India

**Keywords:** Photoacoustic imaging, Image quality assessment, Full-reference metrics, No-reference metrics, Deep learning, Image quality prediction

## Abstract

Photoacoustic (PA) imaging offers high-resolution functional imaging *in vivo*, but image quality varies with acquisition hardware, reconstruction methods, and scanning conditions. Reliable image quality assessment (IQA) is therefore critical for evaluating new PA technologies and ensuring reproducible research. IQA metrics originally proposed for natural images are widely used; however, their relevance to PA imaging has not been systematically benchmarked. We present the first large-scale benchmark of IQA in PA imaging, evaluating 11 full-reference (FR) metrics and 2 no-reference (NR) metrics across nearly one million PA images from five independent datasets. These datasets span phantoms, preclinical small-animal scans, and challenging *ex vivo* and *in vivo* acquisitions with controlled degradations across multiple commercial imaging systems. Metric scores were analyzed statistically to determine which metrics best distinguish differences in image quality. Metrics that showed consistent performance across datasets are modeled using three deep learning architectures (PAQNet, IQDCNN, and EfficientNetIQA), where we trained the models to predict these metric values directly from PA images, enabling automated no-reference quality estimation. The results show that structural similarity-based FR metrics, especially Structural Similarity Index Measure (SSIM) and its variants, reliably capture image quality differences in PAI, whereas conventional metrics like Peak Signal-to-Noise Ratio (PSNR) and existing NR metrics correlate poorly with reconstruction improvements. Deep learning models, particularly PAQNet, achieved high correlations with reference-based scores and provide a practical path to reference-free quality assessment, although generalization across datasets from different systems remains challenging. This study establishes a benchmark for PA image quality evaluation and demonstrates that structure-aware metrics and learned no-reference predictors can enable more reliable and automated quality assessment in PAI. We provide code for IQA metrics evaluation and deep learning models for reproducibility and further development at https://github.com/MellevdB/photoacoustic-image-quality-assessment.git.

## 1 Introduction

Photoacoustic imaging (PAI) combines optical absorption contrast with ultrasonic resolution and penetration, enabling multiscale, multiparametric imaging from microscopy to tomography [1], [2], [3]. PAI has been demonstrated for many clinical applications, yet image quality assessment (IQA) in PAI is not standardized [4], [5]. Most conventional IQA measures, for example, Peak Signal-to-Noise Ratio (PSNR), Structural Similarity Index (SSIM), etc., are originally designed for natural photographs [6], [7] and might not be suitable for diagnostic medical imaging, including PAI [8]. PAI further exhibits modality-specific degradations, for example, limited-view and band-limited artefacts, streaking artefacts from sparse sampling, clutter from out-of-plane signals, sidelobes from beamforming, depth-dependent attenuation, and spectral coloring, which are not well captured by natural-image statistics [9], [10], [11], [12]. Hence, there is a need for IQA methods for PAI.

For PAI, researchers have relied on a mix of signal-based quality measures and conventional IQA metrics. Signal-based measures such as SNR, CNR, contrast ratio, signal-to-background ratio, and spatial resolution are widely used by the PAI community [13], [14]. These are no-reference metrics, but require manual region selection. Inspired by ultrasound imaging, a PAI-specific detectability metric, the generalized contrast-to-noise ratio (gCNR), has gained interest because it avoids dynamic-range and thresholding limitations of classical contrast and CNR, and better reflects target detectability [15]. Multiple works now report gCNR alongside SNR and CNR to quantify visibility gains from beamforming or reconstruction [14], [16]. Although SNR, CNR, and gCNR are considered no-reference metrics, they depend on manual selection of target and background regions. These metrics can be partially automated through region detection algorithms for a specific dataset, but their spatial variation across different target locations makes it difficult to produce a single representative quality score for an image. The need for no-reference metrics that do not rely on manual region selection or reference images is the gap addressed in this work. Palma-Chavez et al. [4] presented a comprehensive review of consensus test methods and current practices in photoacoustic image quality assessment, emphasizing phantom-based evaluation of spatial resolution, geometric accuracy, imaging depth, sensitivity, and contrast. These methods rely on manual region selection and are essential for standardizing imaging systems. Our study complements this work by introducing a framework for automated quality assessment that predicts image quality directly from the reconstructed images without the need for reference data or manual region definition, enabling scalable and objective comparison of image quality across datasets, reconstruction methods, and imaging systems.

At the same time, full-reference evaluation in PAI has been constrained by the scarcity of trusted references *in vivo*. Obtaining a ground truth image for experimental PAI is challenging. However, researchers have developed approaches to overcome this using twin phantoms, controlled targets, and test methods for image quality and oximetry accuracy [17], [18], [19]. This is still limited to a controlled test environment. Several PAI reviews have also focused on clinical PAI and have highlighted the need for IQA practices and the gap between current metrics and clinical needs [4], [5].

Learning-based IQA, which has matured rapidly in natural and medical imaging, offers a route to modality-aware, no-reference quality prediction [6], [7], [20]. In PAI, deep learning has predominantly targeted reconstruction, artefact removal, and denoising under sparse or limited-view sampling [11], [21], [22], [23], [24], with quality judged by a mixture of PSNR, SSIM, and signal-based metrics, and PSNR has been shown not to correlate with expert opinion [8]. Hence, benchmarking existing imaging quality metrics and developing learning-based IQA for PAI for no-reference quality assessment are urgent requirements for clinical translation.

In this work, we first benchmark a broad range of full-reference and no-reference metrics on diverse PAI datasets, including phantoms and *in vivo* images, to identify which metrics best reflect differences in perceptual quality. Second, from the top-performing metrics, we train and analyze learning-based no-reference predictors that map PA images to predict the quality scores. Using nearly one million examples spanning simulated, phantom, *in vivo*, and clinically inspired images, we study generalization, out-of-distribution behavior, and model interpretability. Our contributions are (i) a quantitative comparison of eleven FR and two NR metrics across multiple datasets and acquisition regimes, (ii) three NR-IQA models, PAQNet, IQDCNN, and EfficientNetIQA, trained to mimic the top-ranked metrics and explored in multi-task settings, and (iii) an interpretability analysis via Grad-CAM based analysis to probe what image regions drive quality predictions. Our work is the first on large-scale IQA in PAI with images from multiple commercial systems, targets, and applications, benchmarking the highest number of metrics so far, a step towards learning-based no-reference quality assessment, and providing an open-source resource to the community.

## 2 Datasets

To capture a wide range of image qualities and artefacts, we compiled several PAI datasets spanning simulations, phantoms, small animal imaging, and human-inspired scenarios. Each dataset includes reference “high-quality” reconstructions and corresponding lower-quality images produced by altering imaging parameters (such as number of detectors, view angle, or Signal-to-Noise Ratio). Below we outline each dataset and its key characteristics.

### 2.1 OADAT dataset

We utilized the *OADAT* open-access collection [25], which provides both experimental and synthetic optoacoustic data. In particular, we used from the OADAT dataset:

• **Simulated Cylinders Dataset (SCD):** This consists of simulated tomographic reconstructions using a circular transducer geometry (full 360° view). This also includes variants including a full-view reference and a sparse-view reconstruction with fewer detection angles (e.g., half-circle or quarter-circle coverage). The sparse configurations introduce angular undersampling artefacts.
• **Multispectral Forearm Dataset (MSFD):** This configuration consists of a multi-segment arc transducer geometry, with data from forearm imaging [25]. This dataset contains multiple wavelength acquisitions (700–850 nm) but for our purposes we use a representative wavelength (760 nm) to focus on spatial quality. The reconstructions are available for a full multisegment array (reference) and sparser sampling configurations (e.g., using only a subset of segments), leading to spatial sparsity artefacts. The MSFD subset is one of the largest datasets, contributing to the order of $10^{5}$ reconstructed images.
• **Single Wavelength Forearm Dataset (SWFD):** This consists of a simulated linear-array geometry with a wide field of view, including both a full-view detector reference and sparser versions (e.g., skipping detectors or reducing aperture). We utilized both a multi-segment (ms) linear configuration and a single-segment (or semi-circular, sc) configuration [25]. These allow testing metric sensitivity to different coverage; the semi-circular case is particularly challenging as the limited view induces significant reconstruction blurring in the reconstructed images.

All OADAT-sourced images are reconstructed optoacoustic tomographic slices with known ground truth references (the full-sampling or full-view cases). The simulated data has the advantage of a very large number of images (hundreds of thousands across parameter variations), facilitating robust statistics. We applied preprocessing to these datasets, including bandpass filtering (0.1–6 MHz) and intensity normalization [25], [26], [27], to ensure consistency before metric computation. All images were reconstructed using a delay-and-sum backprojection algorithm with a fixed speed-of-sound, following the standard reconstruction pipeline provided with the dataset.

### 2.2 Experimental phantom and *in vivo* data

We also utilized PAI data from controlled phantom experiments and small animal imaging, which was captured using a tomography system (full-ring 512-element transducer).

• The Phantom dataset consists of tissue-mimicking phantoms with embedded structures (e.g., printed vascular patterns on polyurethane films) imaged in a water tank. Reference images were acquired with a full 512-element ring for 360° coverage, and degraded versions were generated by using only a subset of the detection elements (e.g., 8, 16, 32, …, 128 evenly spaced detectors) to simulate sparse-sampling reconstructions [28]. As expected, image quality improves as more detectors are used (sparse8 being poorest, sparse128 closer to reference) [28].
• A Virtual Phantom dataset was similarly created, but using simulated data of a digital phantom through the same ring geometry. The Virtual Phantom (“V Phantom”) helps examine whether trends observed in real phantom data persist in an idealized simulation of a phantom [28].
• The Mice dataset consists of *in vivo* whole-body mouse scans [28]. Mice were imaged with the full ring array (providing a ground truth reconstruction) and with progressively sparser angular sampling. We denote reconstructions by the number of virtual projections (e.g., sparse4, sparse8, …, sparse256) used in the image formation [28]. This dataset presents realistic biological variability (heterogeneous tissues, blood vessels, etc.) and thus tests metric performance under more complex, natural image features. For instance, deeper structures and higher noise in mice images pose challenges that may differ from the phantom case.

All experimental data were preprocessed similarly to simulations (bandpass filtered and min–max normalized per image). For the *in vivo* data, we ensured any irrelevant borders or imaging artefacts (e.g., coupling medium boundaries) were cropped out prior to analysis, so that metrics focus on the actual anatomical region of interest. Following the reconstruction pipeline provided with the dataset, images in the Phantom and *in vivo* mouse datasets were reconstructed using a universal backprojection algorithm. Prior to reconstruction, the raw signals were bandpass filtered. For *in vivo* mouse imaging, the reconstruction was adapted to account for a heterogeneous speed-of-sound distribution between the animal and the surrounding coupling medium, while for phantom experiments a uniform speed of sound was assumed.

### 2.3 Experimental frame averaging (EFA) dataset

To assess performance under noise-induced degradation, we used the Experimental Frame Averaging (EFA) dataset [29]. The dataset is acquired with the handheld LED-based AcousticX system (Cyberdyne Inc., Tsukuba, Japan), which integrates a pulsed 850 nm LED array and a 128-element linear ultrasound transducer (center frequency ∼7 MHz) in a handheld probe configuration [30]. Image quality was varied by changing the number of frame averages during acquisition: fewer averages yielded lower Signal-to-Noise Ratio (SNR) with stronger noise, while higher averaging produced smoother, high-SNR reference images.

The dataset includes five phantoms and three **in vivo** human extremity scenes:

• **Phantoms:** Vascular network phantoms with vessel-like structures, one Derenzo resolution test pattern, and one angular sensitivity test pattern. Each target was ink-printed on 60 µm polyurethane films [17].
• ***In vivo*:** Images of human extremities (e.g., fingers, arms) highlighting vascular structures [9].

Each phantom and **in vivo** target was acquired at seven frame-averaging levels: 25 600 (used as reference), 128, 256, 384, 640, 1280, and 2560 frames, referred to as PA1 to PA7. Thus, the dataset provides controlled degradations in SNR and speckle appearance while preserving anatomical or phantom structure.

Prior to metric computation, all EFA images were intensity- normalized, and uniform background regions (e.g., above the tissue surface) were cropped to prevent bias. This dataset is particularly suited for evaluating how well metrics capture quality degradation due to noise and averaging. Images in the EFA dataset were reconstructed using a Fourier-domain reconstruction method followed by envelope detection [31].

### 2.4 Neurovascular network explorer (NNE) dataset

The NNE dataset consists of high-resolution 2-photon vessel diameter measurements from the mouse S1 cortex. In this study, the data were used to generate simulated and experimental photoacoustic (PA) images for fluence compensation analysis. Using the AcousticX LED-based imaging system and digital phantom printing onto polyurethane films, the dataset includes realistic reconstructions with ground truth acquired in both water and tissue-mimicking media. Images were reconstructed under various noise conditions (10–50 dB), enabling the evaluation of deep learning-based models [32]. NNE images were reconstructed using time reversal reconstruction approach followed by envelope detection [33].

Across all datasets, we set aside appropriate training, validation, and testing splits for the deep learning experiments (as detailed in Section 3). In total, our compiled data spans on the order of $10^{6}$ images, making this one of the largest studies of PAI image quality to date.

## 3 Methods

In this section, we describe the methods for assessing image quality in PAI, including the definitions of the image quality metrics, the statistical evaluation approach for metrics, and the design and training of deep learning models for quality prediction. We also outline the experimental protocol for model training, validation, and testing.

### 3.1 Image quality metrics

We evaluated a broad set of established IQA metrics, covering full-reference metrics, which require a ground truth image for comparison, and no-reference metrics, which operate on the image alone. All metrics are computed on a per-image basis. For full-reference metrics, each reconstructed image from a dataset was compared to its corresponding high-quality reference image, considered as the “ground truth”. For no-reference metrics, the score is computed from the reconstructed image itself. First, we will discuss the FR metrics in detail.

#### 3.1.1 Full-reference (FR) metrics

To establish a benchmark for assessing image fidelity, reference-based metrics are used to compare the reconstructed images to the ground truth. Eleven image quality metrics are used to evaluate the PA images. All full-reference metric computations are performed using the PyTorch Image Quality (PIQ) library [26], [27], a modern and GPU-compatible package for reproducible image quality assessment in deep learning environments. The metrics *S3IM*, *UQI*, and *FSIM* are not implemented in PIQ and were therefore computed using custom implementations based on their published formulations.

##### **PSNR**

Peak Signal-to-Noise Ratio (PSNR) will quantify the difference between the reference image (x) and the reconstructed image (y) by comparing the maximum possible signal power to the power of corrupting noise. PSNR is defined as :

$$\text{PSNR}=10\cdot \log_{10}\left(\frac{{\text{MAX}}^{2}}{\text{MSE}}\right)$$

where MAX is the maximum possible pixel value of the image (defined by the data range) and MSE is defined as:

$$\text{MSE}=\frac{1}{N}\overset{N}{\underset{i=1}{\sum }}{\left(x\left[i\right]-y\left[i\right]\right)}^{2}$$

where N is the total number of elements (pixels) in the images. x[i] and y[i] are the intensity values of the ith pixel in images x (reference) and y (reconstructed), respectively.

A higher PSNR score indicates a higher quality of the reconstructed image compared to the ground truth, indicating less noise in the input image [34].

##### **SSIM**

Structural Similarity Index Measure (SSIM) will be used to measure the structural similarity between the reconstructed photoacoustic image and the ground truth, focusing on luminance, contrast, and structure. SSIM is defined as:

$$\text{SSIM}\left(x,y\right)=\frac{\left(2\mu_{x}\mu_{y}+C_{1}\right)\left(2\sigma_{xy}+C_{2}\right)}{\left(\mu_{x}^{2}+\mu_{y}^{2}+C_{1}\right)\left(\sigma_{x}^{2}+\sigma_{y}^{2}+C_{2}\right)}$$

where $\mu_{x}$ and $\mu_{y}$ are the mean intensity of images x and y, respectively. $\sigma_{x}^{2}$ and $\sigma_{y}^{2}$ are the variance of images x and y, respectively. $\sigma_{xy}$ is the covariance of images x and y. $C_{1}={\left(K_{1}R\right)}^{2}$ is a stabilizing constant to avoid division by zero, derived from $K_{1}$ (default 0.01) and R (data range). $C_{2}={\left(K_{2}R\right)}^{2}$ is also a stabilizing constant to avoid division by zero, derived from $K_{2}$ (default 0.03) and R (data range) [35], [36].

##### **MS-SSIM**

Multi-Scale Structural Similarity (MS-SSIM) extends SSIM by comparing image structures at multiple spatial resolutions. It decomposes the image into different scales using low-pass filtering and downsampling, combining luminance, contrast, and structure comparisons at each level. MS-SSIM is defined as:

$$\text{MS-SSIM}\left(x,y\right)={\left[l_{M}\left(x,y\right)\right]}^{\alpha_{M}}\overset{M}{\underset{j=1}{\prod }}{\left[c_{j}\left(x,y\right)\right]}^{\beta_{j}}{\left[s_{j}\left(x,y\right)\right]}^{\gamma_{j}}$$

where M is the number of scales, and l, c, and s represent luminance, contrast, and structure comparisons respectively. MS-SSIM improves robustness against resolution and viewing condition changes [37].

##### **IW-SSIM**

Information-Weighted SSIM (IW-SSIM) weights the local SSIM scores by the local information content, giving more importance to regions with higher entropy. It aims to reflect human visual sensitivity to structural information by emphasizing perceptually important regions during averaging [38].

##### **Sparse SSIM (S3IM)**

S3IM evaluates structural similarity only within informative regions rather than across the entire image. This is achieved by generating adaptive masks that highlight vascular or edge-like structures, while suppressing homogeneous background regions that are less informative in photoacoustic images. The method can be described in three steps:

1. Adaptive mask generation. For both the reference and predicted image, a Gaussian local thresholding method is applied using a neighborhood size proportional to the image resolution:
  $$\text{neighborhood\_size}=\frac{H}{16}\times 2+1,$$
  where H is the image height. A sensitivity parameter (offset=0.5) controls the threshold level. This creates binary masks (${\text{mask}}_{1},{\text{mask}}_{2}$) that mark significant structures (e.g., vessels, high-contrast regions).
2. Mask combination and application. The masks from the reference and predicted image are combined using a logical OR, ensuring that any structure present in either image is included. The resulting mask is applied to both images, zeroing out background pixels:
  $$I^{\prime }=I\cdot \text{mask}.$$
3. SSIM computation on masked regions. The standard SSIM map is computed between the masked images. The S3IM value is then obtained as the mean SSIM restricted to the masked regions:
  $$\text{S3IM}=\frac{\sum \left({\text{SSIM}}_{\text{map}}\cdot \text{mask}\right)}{\sum \text{mask}}.$$

By focusing exclusively on sparse, informative regions, S3IM reduces the influence of noise-dominated background and improves sensitivity to structural fidelity. This is particularly relevant for PA images, where vascular structures occupy only a small fraction of the image domain [39].

##### **HAARPSI**

Haar Perceptual Similarity Index (HaarPSI) leverages Haar wavelets to model the early stages of human visual processing. It computes local similarities in the wavelet domain and aggregates them with perceptual weights. HAARPSI is defined as:

$$\text{HaarPSI}\left(x,y\right)=\sigma \left(\frac{\underset{i}{\sum }w_{i}\cdot {\text{sim}}_{i}}{\underset{i}{\sum }w_{i}}\right)$$

where σ is a sigmoid function and ${\text{sim}}_{i}$ is the local similarity in Haar bands. HaarPSI is designed for high correlation with human perception at low computational cost [40].

##### **FSIM**

Feature Similarity Index (FSIM) will be used to assess the perceptual similarity between the reconstructed photoacoustic image and the ground truth, focusing on phase congruency and gradient magnitude to capture structural and textural details. FSIM is defined as:

$$\text{FSIM}=\frac{\underset{i,j}{\sum }S_{L}\left(i,j\right)\cdot \max\left({\text{PC}}_{1}\left(i,j\right),{\text{PC}}_{2}\left(i,j\right)\right)}{\underset{i,j}{\sum }\max\left({\text{PC}}_{1}\left(i,j\right),{\text{PC}}_{2}\left(i,j\right)\right)}$$

where $S_{L}\left(i,j\right)=\left(S_{\text{PC}}{\left(i,j\right)}^{\alpha }\cdot S_{\text{G}}{\left(i,j\right)}^{\beta }\right)$ is the combined similarity measure of phase congruency (PC) and gradient magnitude (GM). $S_{\text{PC}}\left(i,j\right)$ is the phase congruency similarity between the original and predicted images at pixel (i,j). $S_{\text{G}}\left(i,j\right)$ is the gradient magnitude similarity between the original and reconstructed images at pixel (i,j). ${\text{PC}}_{1}\left(i,j\right),{\text{PC}}_{2}\left(i,j\right)$ are the phase congruency maps of the original and reconstructed images, respectively. α,β are the weights for the relative importance of $S_{\text{PC}}$ and $S_{\text{G}}$ (default is 1). $T_{1},T_{2}$ are stabilizing constants for $S_{\text{PC}}$ and $S_{\text{G}}$, respectively [41], [42].

##### **GMSD**

Gradient Magnitude Similarity Deviation (GMSD) uses image gradients to assess structural differences. It calculates a pixel-wise similarity map between gradients and uses its standard deviation as the quality score. GMSD is defined as:

$$\text{GMSD}\left(x,y\right)=\text{std}\left(\frac{2G_{x}G_{y}+c}{G_{x}^{2}+G_{y}^{2}+c}\right)$$

where $G_{x}$ and $G_{y}$ are gradient magnitudes of the reference and reconstructed images. A lower GMSD implies higher similarity [43].

##### **MS-GMSD**

Multi-Scale GMSD (MS-GMSD) extends GMSD to multiple resolutions. Similar to MS-SSIM, it averages GMSD scores across scales to better capture human visual perception over various image details and distortions [43].

##### **VIF**

Visual Information Fidelity (VIF) will be used to evaluate the amount of visual information preserved in the reconstructed photoacoustic image compared to the ground truth, focusing on the human visual system’s sensitivity to signal fidelity and noise. VIF is defined as:

$$\text{VIF}=\frac{\underset{i}{\sum }\underset{j}{\sum }g_{ij}\cdot \log\left(1+\frac{\sigma_{ij}^{2}}{\sigma_{n}^{2}}\right)}{\underset{i}{\sum }\underset{j}{\sum }\log\left(1+\frac{\sigma_{ij}^{2}}{\sigma_{n}^{2}}\right)}$$

where $g_{ij}$ is a gain factor that represents the relation between the original and reconstructed image pixel values for pixel (i,j). $\sigma_{ij}^{2}$ is the variance of the image signal at pixel (i,j). $\sigma_{n}^{2}$ is the variance of the visual noise (default value is 2). The numerator represents the information fidelity of the distorted image. The denominator represents the information fidelity of the original image [44].

##### **UQI**

Universal Image Quality Index (UQI) will be used to evaluate the similarity between the reference image (x) and the reconstructed photoacoustic image (y), focusing on luminance, contrast, and structural distortions using a localized sliding window approach. UQI is defined as:

$$\text{UQI}=\frac{4\cdot \left(\mu_{x}\mu_{y}\right)\cdot \sigma_{xy}}{\left(\mu_{x}^{2}+\mu_{y}^{2}\right)\cdot \left(\sigma_{x}^{2}+\sigma_{y}^{2}\right)}$$

where $\mu_{x}$ and $\mu_{y}$ are the local means of x and y, respectively, calculated over a sliding window of size ws. $\sigma_{x}^{2}$ and $\sigma_{y}^{2}$ are the local variances of x and y, respectively, also computed within the sliding window. $\sigma_{xy}$ is the local covariance between x and y. The numerator $4\cdot \left(\mu_{x}\mu_{y}\right)\cdot \sigma_{xy}$ combines luminance similarity $\left(\mu_{x}\mu_{y}\right)$ and contrast similarity $\left(\sigma_{xy}\right)$, capturing the joint distribution of the two images. The denominator $\left(\mu_{x}^{2}+\mu_{y}^{2}\right)\cdot \left(\sigma_{x}^{2}+\sigma_{y}^{2}\right)$ normalizes the metric to account for scaling, ensuring UQI is bounded between −1 and 1, with values closer to 1 indicating higher similarity [45].

#### 3.1.2 No-reference (NR) metrics

Next, we will discuss the NR metrics utilized in this work.

##### **BRISQUE**

Blind/Referenceless Image Spatial Quality Evaluator (BRISQUE) is a no-reference image quality assessment metric that evaluates the perceptual quality of an image based on natural scene statistics (NSS) in the spatial domain. Unlike full-reference methods, BRISQUE does not require a ground truth image for comparison. Instead, it analyzes the image’s statistical properties to predict distortions such as noise, blur, and compression artefacts. BRISQUE is computed in three main steps:

1. Mean Subtracted Contrast Normalization (MSCN): The image is processed to remove local mean intensity variations and normalize contrast:
  $$\overset{ˆ}{I}\left(i,j\right)=\frac{I\left(i,j\right)-\mu \left(i,j\right)}{\sigma \left(i,j\right)+C}$$
  where μ(i,j) is the local mean, σ(i,j) is the local standard deviation, and C is a small constant to avoid division by zero.
2. Pairwise Product Computation: Four directional spatial relationships are computed to capture structural distortions:
  $$H\left(i,j\right)=\overset{ˆ}{I}\left(i,j\right)\cdot \overset{ˆ}{I}\left(i,j+1\right)$$
  $$V\left(i,j\right)=\overset{ˆ}{I}\left(i,j\right)\cdot \overset{ˆ}{I}\left(i+1,j\right)$$
  $$D1\left(i,j\right)=\overset{ˆ}{I}\left(i,j\right)\cdot \overset{ˆ}{I}\left(i+1,j+1\right)$$
  $$D2\left(i,j\right)=\overset{ˆ}{I}\left(i,j\right)\cdot \overset{ˆ}{I}\left(i+1,j-1\right)$$
3. Feature Extraction and Quality Prediction: Statistical features are extracted from the MSCN coefficients and pairwise product images. These features are fitted to a generalized Gaussian distribution (GGD) and an asymmetric generalized Gaussian distribution (AGGD) to form a 36-dimensional feature vector. The final BRISQUE score is obtained by feeding these features into a support vector machine (SVM) trained on human-labeled image quality datasets.

BRISQUE produces a quality score in the range of 0 to 100, where lower values indicate higher image quality and higher values suggest a more distorted image. This metric is useful for assessing real-world image degradations without requiring a reference image [46].

##### **CLIP-IQA**

CLIP-IQA is a recent no-reference image quality assessment method that leverages the contrastive vision-language representations of the CLIP model. Instead of computing traditional quality scores, CLIP-IQA uses image-text similarity to align degraded images with textual quality descriptions. The method involves embedding both the image and a set of predefined quality-related text prompts using CLIP, and computing their cosine similarity:

$$\text{CLIP-IQA}\left(I\right)=\underset{t\in T}{\max}\cos\left(\phi \left(I\right),\phi \left(t\right)\right)$$

where ϕ(⋅) denotes the CLIP embedding function, I is the input image, and T is the set of textual prompts (e.g., “This is a high-quality image”). This approach allows CLIP-IQA to generalize across multiple distortion types without requiring a reference image or specialized training on IQA datasets. It has shown competitive performance in both full-reference and no-reference IQA settings [47].

#### 3.1.3 Region-based detectability metrics

To complement the full-reference and no-reference analyses, we computed region-based detectability metrics, namely Signal-to-Noise Ratio (SNR), Contrast-to-Noise Ratio (CNR), and generalized CNR (gCNR), on representative reconstructions from multiple datasets (Mice, Phantom, SWFD_sc, SWFD_ms). For each image, three signal regions of interest (ROIs) were placed on three visible targets, and three corresponding local background ROIs of identical size were placed adjacent to each target. The mean and standard deviation were computed for each region. All region-based metrics were computed on beamformed images in linear scale. The SNR was defined as [4]:

$$\mathrm{SNR}\;=\;\frac{S}{\sigma_{b}},$$

where S is the mean pixel value within a target ROI, and $\sigma_{b}$ is the standard deviation of pixel values within the paired background ROI.

We report **CNR** as:

$$\mathrm{CNR}\;=\;\frac{\left|\mu_{s}-\mu_{b}\right|}{\sigma_{b}},$$

where $\mu_{s}$ is the mean pixel value within a signal ROI, and $\mu_{b}$ and $\sigma_{b}$ are the mean and standard deviation within the paired background ROI.

Generalized CNR (**gCNR**) quantifies the separability of signal and background pixel-value distributions and is robust to dynamic-range and thresholding effects [15]. Let $p_{s}$ and $p_{b}$ denote the normalized histograms of the signal and background ROIs, estimated with a fixed number of bins over the joint pixel-value range of the paired ROIs. gCNR is given by:

$$\mathrm{gCNR}\;=\;1-\underset{i}{\sum }\min\;\left(p_{s}\left(i\right),\;p_{b}\left(i\right)\right),$$

which equals 1 when the distributions do not overlap and 0 when they are identical.

For each image, metrics were computed for each of the three signal background pairs and then averaged to obtain one SNR, one CNR, and one gCNR per image.

### 3.2 Statistical evaluation of metrics

We evaluated each metric based on its ability to distinguish between reconstruction qualities under varying acquisition and processing settings. Known configuration differences within each dataset (e.g., 32 vs. 128 detectors, or 128 frame vs. 25600 frame averaging) were used as ground-truth ordinal relationships, where the configuration with more detectors or greater averaging was expected to yield higher quality. We assume that increasing the number of transducers and the amount of frame averaging leads to higher photoacoustic image quality. Our primary goal is to test whether an IQM can reliably separate these predefined quality levels [48]. This should be regarded as a surrogate for human observer studies, not a replacement for direct assessment of perceived image quality or diagnostic performance. Using these pairings, we performed the following evaluations:

• **Pairwise significance testing:** For each configuration pair within a dataset (e.g., sparse32 vs. sparse128 in Phantom, or 2-frame vs. 7-frame in EFA), we applied a two-sided t-test to the metric values. Differences were considered significant at p<0.05.
• **Significant Count (SC):** For each metric, we counted the number of image subset pairs with statistically significant differences. A higher count indicates greater sensitivity to quality variations.
• **Normalized Mean Difference (NMD):** For significant comparisons, we computed the absolute difference in mean metric values, normalized by the metric’s value range, followed by computing the mean value. This quantifies effect size, i.e., how strongly the metric separates reconstruction qualities on average.
• **Composite Score (CS):** Finally, we defined a composite score as the product of the Significant Count and the Normalized Mean Difference:
  CS=SC×NMD.
  This provides a unified ranking criterion, favoring metrics with both high sensitivity and strong effect size.

In addition to the formal tests, we examined metric distributions via boxplots and their correlations with each other to see if some metrics are redundant. Where appropriate, we also computed Spearman’s rank correlation between metric scores and the known “quality level” (such as number of detectors) as another check on monotonic behavior.

For all metrics, the absolute value of the normalized mean difference was used, ensuring that metrics where lower values indicate higher image quality (e.g., GMSD) are treated equivalently to metrics whose values increase with quality. Hence, composite scores quantify sensitivity to quality changes independent of metric directionality.

All statistical analyses were performed using Python (SciPy and pandas libraries). Given the very large sample size (>100k images for some metrics), even small differences often yield significant p-values; therefore, our focus was on patterns of consistency and effect sizes rather than just significance.

### 3.3 Deep learning models for quality prediction

We also designed three deep learning models to predict image quality from PA images. Each model takes a single reconstructed PA image (grayscale, 128 × 128 pixels after preprocessing) as input and outputs one or more scalar quality scores for the image metric. During training, these models were supervised with “ground truth” quality values provided by one or multiple FR metrics. In essence, the networks learn to mimic a given image quality metric, enabling no-reference prediction of that metric. The three models evaluated in this work are described below. PAQNet is newly proposed in this work, while IQDCNN and EfficientNetIQA are adapted for PA imaging but have not been used for this purpose before.

#### 3.3.1 PAQNet

We developed a custom convolutional neural network specifically for this task. It consists of four convolutional layers with increasing filters (32, 64, 128, 256) and 5 × 5 or 3 × 3 kernels, each followed by ReLU activation and 2 × 2 max pooling. This is followed by two fully connected layers (128 units each with ReLU) and a final linear output node for regression. The model has around 2.5 million parameters, making it relatively lightweight. We initialized weights randomly and optimized the design via extensive hyperparameter search and K-fold cross-validation on the training set. PAQNet was trained using a MAE loss and ADAM optimizer with learning rate of $1e^{-4}$.

Fig. 1Deep learning architectures used for photoacoustic image quality prediction. (a) Proposed PAQNet, a lightweight custom CNN. (b) IQDCNN, adapted from prior medical NR-IQA work [48]. (c) EfficientNetIQA, based on EfficientNet-B0 with a regression head.Fig. 1

**(a):**
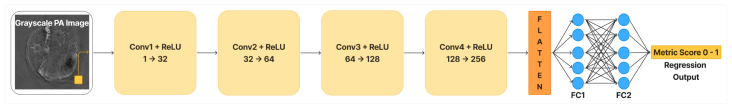
PhotoacousticQualityNet (PAQNet): 4×Conv+ReLU with pooling, flatten, two FC layers, and a scalar regression output.

**(b):**
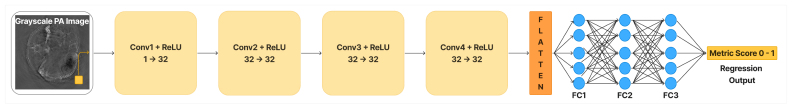
IQDCNN: 4×Conv layers (32 filters, 5 × 5), followed by 3 FC layers (1024 units, dropout 0.3), and regression output.

**(c):**
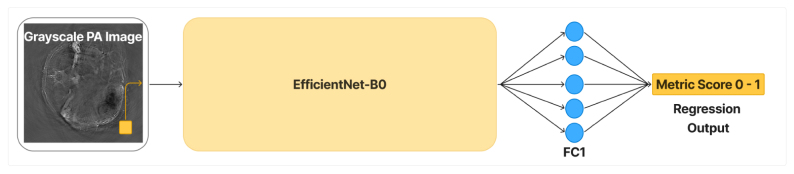
EfficientNetIQA: EfficientNet-B0 backbone with modified first layer (grayscale input) and regression head (128-unit FC + output node).

#### 3.3.2 IQDCNN

This is a deep CNN model adapted from a prior no-reference IQA approach for medical images [48]. The architecture is a deep network with four convolutional layers (each 32 filters of size 5 × 5) followed by three fully connected layers of 1024 units. We use ReLU activations and dropout (rate 0.3) on the fully connected layers (see Fig. 1). IQDCNN was originally proposed for MRI quality assessment [48], and we repurposed it for PAI. It has around 3.8 million parameters. We trained it with an MAE loss (absolute error) and Adam optimizer, learning rate of $1e^{-4}$. IQDCNN serves as a baseline deep regression model for comparison with the proposed model.

#### 3.3.3 EfficientNetIQA

This is a model based on EfficientNet-B0 [49], a modern convolutional network architecture known for its efficiency and strong performance on image tasks. We modified EfficientNet-B0 to accept single-channel input (by averaging the weights of the initial RGB filters) and replaced the top classification layers with a regression head. The regression head consists of a fully connected layer with 128 units (ReLU activation) and an output node (see Fig. 1). EfficientNetIQA has roughly 4.2 million parameters, the largest of the three models, and a significantly deeper feature extraction part (with many more layers than the custom CNNs). We used the Adam optimizer with a learning rate of $1e^{-4}$ and an MAE loss for training. This model was expected to capture more complex feature relationships for quality prediction, potentially improving accuracy at the cost of more computation.

#### 3.3.4 Training procedure and evaluation protocol

All models were implemented in PyTorch. We trained each model to predict specific metric scores. In our primary experiments, we trained separate instances of the models for each of five representative FR metrics (SSIM, HAARPSI, IW-SSIM, S3IM, and GMSD). These metrics were chosen to span different types (structural, perceptual, gradient-based) and because they showed strong reliability in our metric benchmark (see Section 4, Results). For example, a “PAQNet-SSIM” model was trained using SSIM scores of images as ground truth. The models thus learn a mapping from an input image to the quality score that a given metric would assign.

We also explored training models to predict multiple metrics simultaneously (multi-output models, described below) as an ablation to see if joint learning helps in better prediction of the IQMs. The dataset was split into training, validation, and test sets with careful consideration to avoid overfitting and ensure robust evaluation. Datasets with a large number of images, such as MSFD and SWFD (multisegment), were used for training to provide a rich and diverse learning set. Smaller or more specialized datasets, such as NNE (Denoising) and V-Phantom, were assigned to the validation set to support reliable hyperparameter tuning without overlapping with training data.

To maintain a strict separation between training and evaluation, several datasets were excluded entirely from both training and validation. These include SCD (both multisegment and virtual circle configurations), SWFD (semicircle), Mice, and the PA Experiment dataset (EFA), which were reserved exclusively for testing. This strategy enables the evaluation of model generalization to unseen datasets and imaging conditions.

Importantly, evaluation is performed separately per dataset rather than on the combined test set. This allows for a more nuanced analysis of model performance across different data characteristics and acquisition setups. The use of distinct datasets for training and validation was a deliberate design choice to test cross-domain generalization. OADAT contains substantially more samples and many highly similar consecutive slices, so using it for both training and validation would risk information leakage and overly optimistic performance estimates. Under this protocol, validation loss is used to monitor trends and detect overfitting rather than to provide an in-domain performance measure (see Table 1).

We performed K-fold cross-validation (with K=5) on the training set when tuning hyperparameters for PAQNet. For IQDCNN and EfficientNetIQA, we adopted hyperparameters from literature or initial trials and then made minor adjustments based on validation performance. Early stopping was employed based on validation loss, to avoid overfitting.

**Table 1:** Image counts per dataset used for training, validation, and testing in the deep learning experiments.

Dataset (Split)	Number of images
**Train**	

MSFD	453,600
SWFD (ms)	156,912
Phantom	2345

**Total Train**	**612,857**

**Validation**	

NNE (Denoising)	25,420
V-Phantom	490

**Total Validation**	**25,910**

**Test**	

SCD (vc + ms)	160,000
SWFD (sc)	156,912
Mice	1918
EFA	711

**Total Test**	**319,541**

During training, images were fed in batches of 16. The training was run on NVIDIA A100 GPUs (40 GB memory); each model took on the order of a few hours to train until convergence when using ∼612k training images. We utilized the PyTorch Lightning framework for streamlined training and checkpointing.

Each trained model was evaluated on the test set by computing the mean absolute error (MAE) between predicted and true metric values, as well as the Spearman ρ and Pearson r correlation coefficients. These statistics were computed for each dataset in the test set to quantify how well the model predicts quality in seen vs. unseen domains. In particular, we report performance on (i) the OADAT-derived test images (which are similar in distribution to much of the training data), (ii) the Phantom and Mice images (somewhat similar but with real experimental variability), and (iii) the EFA images (distinct distribution with LED illumination and high noise, not seen in training).

#### 3.3.5 Multi-output quality prediction

In an ablation experiment, we trained “multi-output” versions of the above models that predict multiple quality metrics at once. The architectures were identical to their single-output counterparts except that the final fully connected layer had n outputs (with n=2 or 5 in our experiments, corresponding to combinations of metrics). For example, a PAQNetMulti model was trained to output both SSIM and GMSD scores simultaneously for each input image. The idea is that by learning to predict two complementary metrics, the model’s internal representation might become more robust and general (e.g., focusing on features relevant to both structural similarity and gradient fidelity). We tried two pairwise combinations (SSIM+GMSD, SSIM+HAARPSI) and one 5-metric combination (SSIM + GMSD + HAARPSI + S3IM + IW-SSIM) to push the limit of joint learning.

During multi-output training, we used a joint loss equal to the average of the individual metric losses (all metrics were normalized to comparable scales for stability). We monitored the validation loss for each output as well as the average. The multi-output models were otherwise trained with the same settings as single-output. After training, we evaluated whether the multi-metric models achieved lower error on each metric compared to models trained on that metric alone.

#### 3.3.6 Grad-CAM based interpretability analysis

We used Gradient-weighted Class Activation Mapping (Grad-CAM) to visualize the regions that influenced each model’s quality predictions. Grad-CAM provides a spatial importance map by weighting the activation maps of the final convolutional layer with the gradients of the target output [50]. We generated Grad-CAM maps across three sparsity levels (sparse32, sparse64, and sparse128) using the same slice. These maps reveal which regions most strongly influence model predictions of image quality.

### 3.4 Computational resources

All training and evaluation experiments were executed on a high-performance computing cluster. Each model training typically utilized a single NVIDIA A100 40 GB GPU. Training the largest model (EfficientNetIQA) on the full training set took roughly 10 h. In total, our study consumed approximately 1200 GPU-hours for training and hyperparameter searches across all models. Inference for a single image is fast (on the order of 1–10 ms for the CNN models, and slightly longer for EfficientNetIQA), indicating that once trained, these models could be deployed for real-time quality monitoring in PAI systems.

## 4 Results

We first present the results of the objective metric evaluation, comparing how well each IQA metric reflects known image quality differences in PAI data (Section 4.1). Next, we report the performance of the deep learning models in predicting quality scores, including both in-distribution and out-of-distribution tests (Section 4.2). We then summarize insights from the ablation studies on multi-metric training and interpretability (Section 4.3).

### 4.1 Benchmarking image quality metrics

**Fig. 2:**
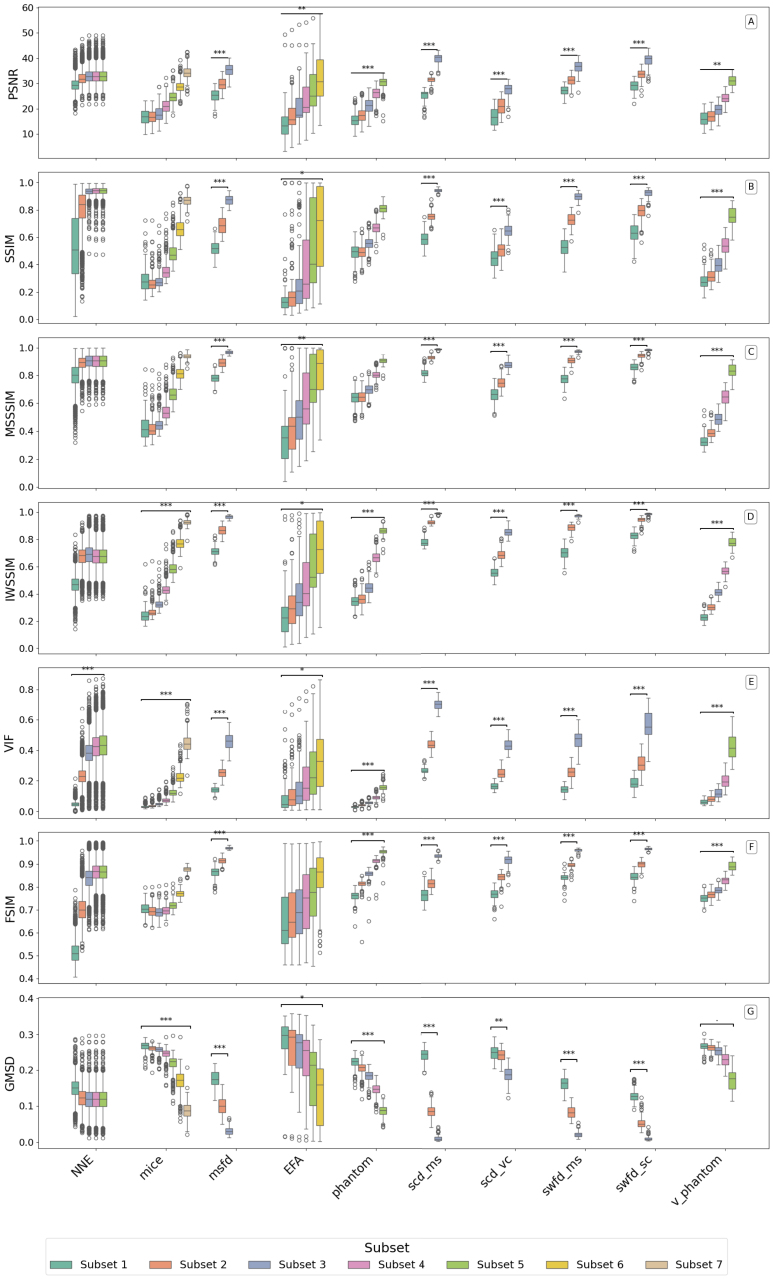
Boxplot of metric distributions (page 1 of 2) on various datasets with subsets representing different configurations or settings resulting in different quality levels.

**Fig. 3:**
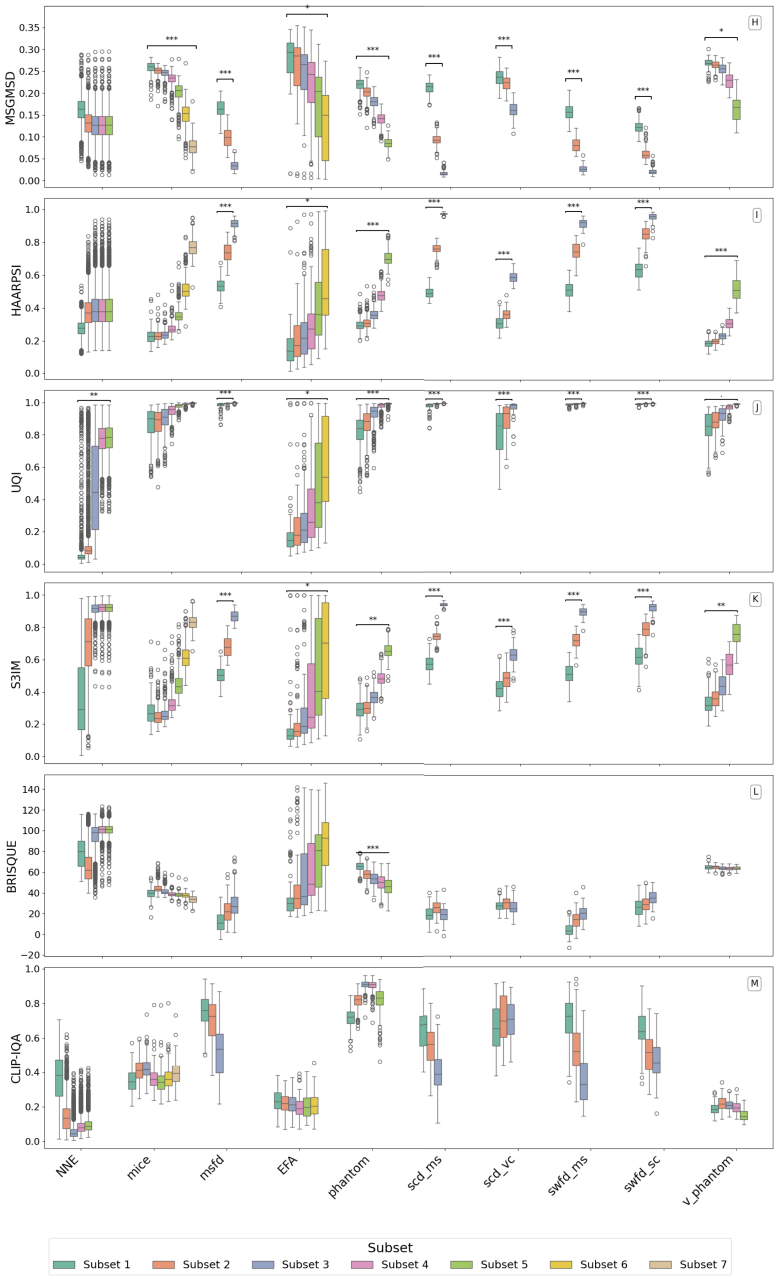
Boxplot of metric distributions (page 2 of 2) on various datasets with subsets representing different configurations or settings resulting in different quality levels.

This section evaluates how well different image quality metrics distinguish between reconstruction configurations or settings across multiple datasets. The metric results are visualized in Fig. 2, Fig. 3, and summarized using a composite ranking in Table 3. In Fig. 2, Fig. 3, each dataset has subsets with different configurations or settings resulting in different quality levels as explained in Table 2. Fig. 2, Fig. 3 plot each IQM across these subset ladders, in the left to right order shown in Table 2. Significant pairwise differences are marked in the figures, and the overall separability is summarized by the composite ranking in Table 3.

**Table 2:** Subset ladders corresponding to the box plots in Fig. 2, Fig. 3. Within each dataset, subsets are ordered from lower to higher expected image quality.

Dataset	Subset ladder (left to right = higher expected quality)
NNE	SNR 10 dB, 20 dB, 30 dB, 40 dB, 50 dB
MICE	sparse4, sparse8, sparse16, sparse32, sparse64, sparse128, sparse256
PHANTOM	sparse8, sparse16, sparse32, sparse64, sparse128
V_PHANTOM	sparse8, sparse16, sparse32, sparse64, sparse128
EFA	PA2, PA3, PA4, PA5, PA6, PA7 (PA1 shown as reference only)
MSFD (OADAT)	sparse32_w760, sparse64_w760, sparse128_w760
SCD_MS (OADAT)	sparse32, sparse64, sparse128
SCD_VC (OADAT)	sparse32, sparse64, sparse128
SWFD_MS (OADAT)	sparse32, sparse64, sparse128
SWFD_SC (OADAT)	sparse32, sparse64, sparse128

**Table 3:** Ranking of image quality metrics on PAI datasets. Metrics are sorted by the composite score (Significant Count × Normalized Mean Diff). FR = full-reference, NR = no-reference. Higher values indicate better ability to distinguish reconstruction quality differences. SC = Significant Count, NMD = Normalized Mean Difference and CS = Composite Score (CS = SC × NMD).

Metric	SC	NMD	CS
S3IM (FR)	84	0.237	19.89
SSIM (FR)	83	0.235	19.48
IW-SSIM (FR)	79	0.241	19.03
GMSD (FR)	82	0.196	16.10
MS-GMSD (FR)	83	0.191	15.83
HAARPSI (FR)	81	0.194	15.73
MS-SSIM (FR)	81	0.186	15.04
VIF (FR)	83	0.159	13.20
UQI (FR)	79	0.134	10.62
FSIM (FR)	74	0.100	7.39
PSNR (FR)	77	0.092	7.06
BRISQUE (NR)	40	0.052	2.09
CLIP-IQA (NR)	24	0.057	1.38

**Table 4:** An example from MSFD dataset showing how SC, NMD, and CS differ between PSNR and SSIM across three quality configurations (ms,ss32 (Low), ms,ss64 (Med.), ms,ss128 (High)). SC = Significant Count, NMD = Normalized Mean Difference and CS = Composite Score (CS = SC × NMD).

	PSNR			SSIM		
Quality level	Low	Med.	High	Low	Med.	High
Mean value	25.202	29.803	35.452	0.518	0.686	0.872

SC (p<0.05)	3/3			3/3		
NMD (avg.)	0.085			0.236		
CS	0.256			0.708		

#### 4.1.1 Metric performance overview

Composite scores from each dataset in Fig. 2, Fig. 3 are computed using the number of times the metric provided statistically significant difference between subsets and the normalized mean differences. The significance count (SC), normalized mean difference (NMD) and composite scores (CS) are provided in Table 3. Table 4 illustrates how the three indicators, SC, NMD and CS capture differences in statistical separability between metrics on MSFD dataset. As a representative example, PSNR and SSIM are compared across three image quality levels (Low, Medium, High). Although both metrics increase as image quality improves, PSNR values overlap substantially between levels, whereas SSIM values are more distinctly separated. Consequently, SSIM detects significant differences in all three pairwise comparisons (SC = 3/3) and shows larger mean shifts (higher NMD), resulting in a higher composite score. This example demonstrates that two metrics can follow the same trend yet differ in how strongly they distinguish adjacent quality levels. A similar analysis applies to other metrics evaluated in this study, where differences in variance and effect size across quality configurations explain variations in their final ranking. We observe that full-reference (FR) metrics dominate the top of the ranking. The best-performing metric overall is S3IM, with the highest composite score of 19.89, closely followed by SSIM (19.48) and IW-SSIM (19.03). These three are SSIM variants, indicating that structural similarity metrics are particularly effective in capturing degradation across reconstruction settings in photoacoustic imaging.

Beyond SSIM variants, the gradient-based metrics GMSD (16.10) and MS-GMSD (15.83) also perform well, ranking fourth and fifth, respectively. These metrics appear to be particularly sensitive to high-frequency structural changes and artefacts, which are common in undersampled or noisy reconstructions. HAARPSI, which approximates perceptual similarity based on wavelet responses, also achieves a reasonably good composite score. Traditional metrics such as PSNR and FSIM show weaker performance, both ranking near the bottom of the FR group. Surprisingly, VIF, despite being theoretically grounded in information fidelity, underperforms relative to more structure-aware metrics.

Among the no-reference (NR) metrics, BRISQUE (2.09) and CLIP-IQA (1.38) rank lowest overall. These metrics show poor ability to detect consistent differences across configurations, suggesting that current NR-IQA methods may not generalize well to the artefact types and modalities present in photoacoustic data.

#### 4.1.2 Region-based detectability results

**Fig. 4:**
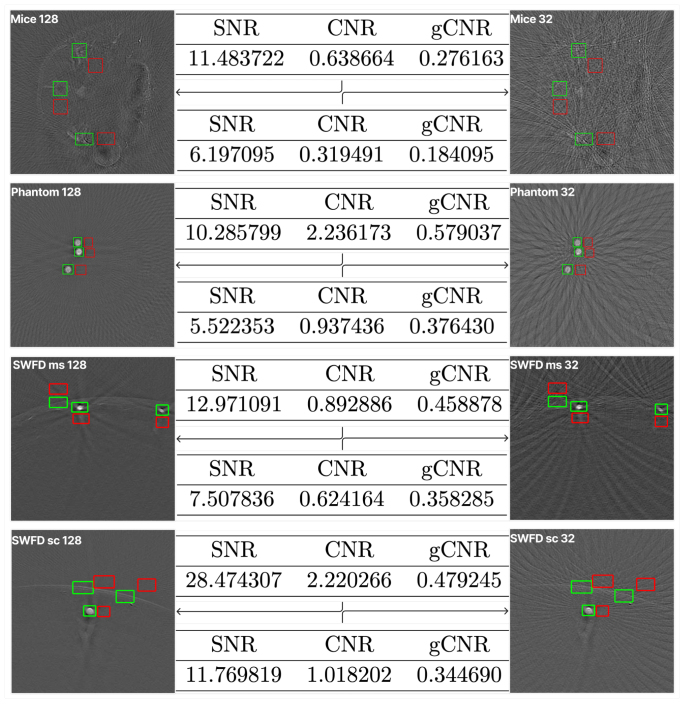
Region-based detectability analysis with manually selected signal (green) and local background (red) ROIs. Numbers report mean (SD in bracket) across images for SNR, CNR, and gCNR. Higher values indicate better target detectability.

Fig. 4 summarizes region-based detectability metrics. Increasing the number of receive channels from 32 to 128 consistently improved SNR, CNR, and gCNR across all datasets. The SNR increased by ＋5.28 for Mice (6.20 vs. 11.48), ＋4.77 for Phantom (5.52 vs. 10.29), ＋5.46 for SWFD_ms (7.51 vs. 12.97), and ＋16.70 for SWFD_sc (11.77 vs. 28.47). Corresponding increases in CNR were ＋0.31, ＋1.30, ＋0.27, and ＋1.20, respectively. The gCNR exhibited smaller increases of ＋0.10, ＋0.20, ＋0.10, and ＋0.14, consistent with its reduced sensitivity to local variance estimates.

#### 4.1.3 Dataset-specific differences

IQM significance varied across the different datasets utilized in this work. For example, on the extremely noisy EFA dataset, most metrics struggled to differentiate frame averaging levels, unless the difference was large; only VIF and UQI consistently detected quality differences at intermediate averaging levels. On the high-quality Phantom and Virtual Phantom datasets, IW-SSIM and HAARPSI had a slight edge, suggesting that those metrics might be particularly attuned to subtle structural differences in cleaner images. Meanwhile, on the complex *in vivo* Mice images, which have more structure, noisy, and differences in reconstruction artefact, SSIM and S3IM showed strong alignment with quality levels with sparse sampling, while metrics like GMSD and MS-SSIM were slightly less correlated. This variability across datasets indicates that while a few top metrics are generally robust, no single metric is perfect for all scenarios.

The top metrics in our study were those capturing structural similarity (SSIM variants) and local perceptual fidelity (HAARPSI, GMSD). These metrics consistently flagged quality improvements across different datasets: for instance, S3IM and SSIM had high sensitivity in the OADAT subsets (MSFD, SCD, SWFD) as well as in Phantom and Mice datasets. In contrast, metrics focusing purely on pixel error (PSNR) or pre-trained natural image statistics (BRISQUE) underperformed, underscoring the importance of structure-aware criteria for PAI. The variance of different image quality metrics motivated our development of learning-based predictors, which can provide top-performing quality metrics in a no-reference way.

**Fig. 5:**
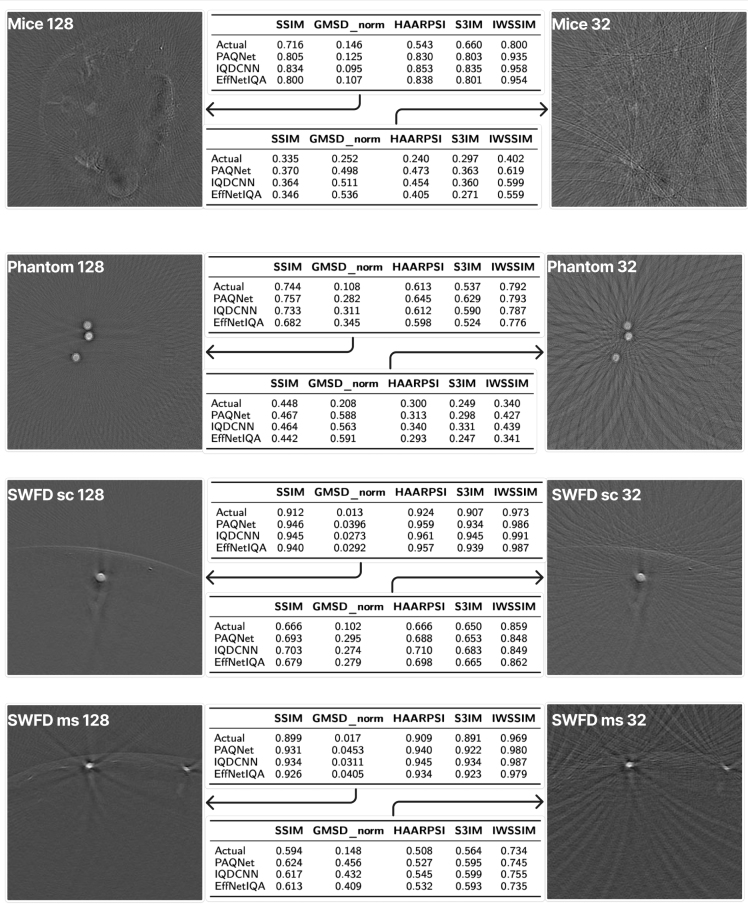
Example test images from different datasets with their corresponding full-reference and predicted metric scores per model.

### 4.2 Deep learning model performance

Five best-performing image quality metrics (IQMs) are modeled using deep learning, and the results are presented in this subsection. We compared full-reference metrics (SSIM, HAARPSI, IW-SSIM, S3IM, and GMSD) across five datasets, evaluated with PAQNet, IQDCNN, and EfficientNetIQA. Fig. 5 shows example images with the corresponding full-reference scores and model predictions, and Table 5 reports mean absolute error (MAE) between predicted and ground-truth scores. Overall, the lowest errors are observed for the OADAT-derived test sets, SWFD_sc and SCD_ms, which are closest to the training distribution, followed by Mice with slightly higher but still low errors. This likely reflects shared acquisition characteristics in OADAT, including similar transducer responses and reconstruction methods, and indicates that the models generalize across applications, target tissue and transducer configurations, when the acquisition settings resemble the training data. In contrast, EFA, acquired with LED illumination and a linear array at a different center frequency, shows markedly higher MAE for all models and metrics, highlighting the challenge of transferring to a distinct imaging setup.

Across models, EfficientNetIQA achieves the best average performance in terms of MAE on HAARPSI (0.1599) and GMSD (0.1421), and remains competitive on SSIM (0.1437) and S3IM (0.1451). It is outperformed on IW-SSIM, where PAQNet (0.1894) and especially IQDCNN (0.1408) achieve lower MAE. PAQNet performs well overall, obtaining the lowest average SSIM error (0.1335) and often ranking among the top two across datasets, for example 0.0248 SSIM MAE on SWFD_sc and 0.0276 on SCD_ms. IQDCNN is strongest on EFA, achieving the lowest MAE for four of the five metrics, including 0.3022 on S3IM and 0.3314 on IW-SSIM.

**Table 5:** Mean Absolute Error (MAE) across image quality metrics and deep learning model (Grouped by Dataset). The highest performing model in bold.

Dataset	Model	SSIM	HAARPSI	IW-SSIM	S3IM	GMSD
Mice	PAQNet	**0.0831**	0.2364	0.2331	**0.0915**	0.2379
	IQDCNN	0.1223	0.2270	0.2246	0.1184	0.2191
	EfficientNetIQA	0.1118	**0.1681**	**0.2060**	0.0974	**0.2116**

SWFD_sc	PAQNet	**0.0248**	0.0368	0.0285	0.0220	0.0313
	IQDCNN	0.0265	0.0358	0.0330	0.0267	0.0353
	EfficientNetIQA	0.0269	**0.0270**	**0.0244**	**0.0193**	**0.0297**

SCD_vc	PAQNet	**0.0779**	0.1841	0.1104	**0.1043**	0.1715
	IQDCNN	0.1195	0.1970	**0.0991**	0.1398	0.1627
	EfficientNetIQA	0.1134	**0.1524**	0.1110	0.1310	**0.1318**

EFA	PAQNet	0.4543	0.4636	0.5591	0.4836	0.5217
	IQDCNN	**0.3808**	**0.3320**	**0.3314**	**0.3022**	0.3745
	EfficientNetIQA	0.4352	0.4100	0.4559	0.4407	**0.2766**

SCD_ms	PAQNet	**0.0276**	0.0490	0.0161	**0.0241**	**0.0465**
	IQDCNN	0.0376	0.0489	0.0161	0.0401	0.0625
	EfficientNetIQA	0.0311	**0.0420**	**0.0133**	0.0371	0.0607

Average	PAQNet	**0.1335**	0.1940	0.1894	0.1451	0.2018
	IQDCNN	0.1373	0.1681	**0.1408**	**0.1254**	0.1708
	EfficientNetIQA	0.1437	**0.1599**	0.1621	0.1451	**0.1421**

Beyond prediction accuracy, we compared the computational footprint of the three deep models. As summarized in Table 6, PAQNet uses substantially fewer parameters and FLOPs than IQDCNN and EfficientNetIQA, and achieves the shortest inference time and lowest peak memory usage under identical hardware and input settings. Combined with the similar MAE reported in Table 5, this confirms that PAQNet provides competitive quality prediction while remaining a lightweight and deployment-efficient option for PAI applications.

**Fig. 6:**
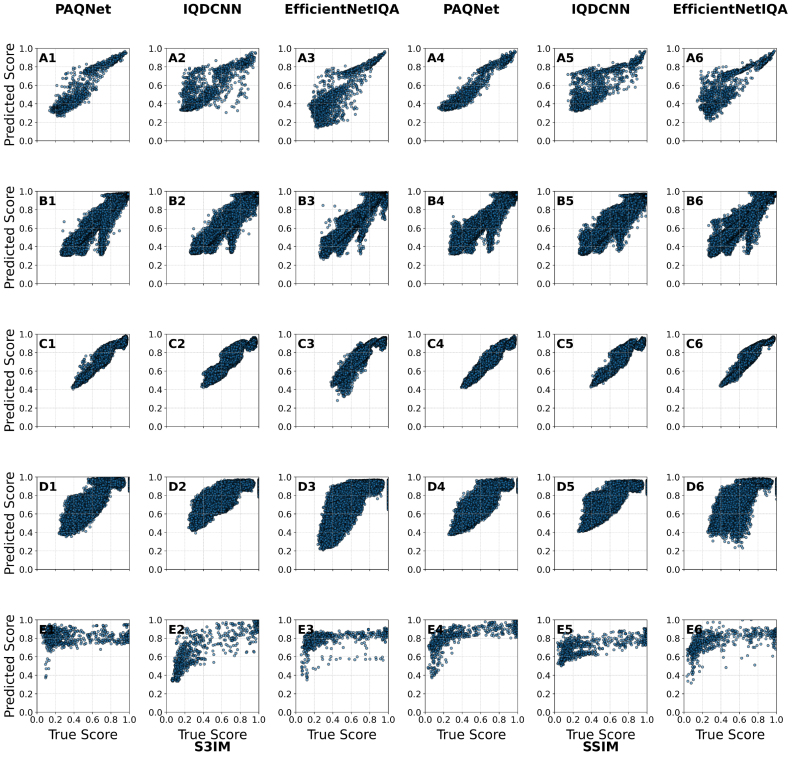
Scatter plots comparing predicted vs. true values for three models (columns) across five datasets (rows). Left side shows **S3IM** results, and right side shows **SSIM** results. Datasets are arranged as: **(A)** Mice, **(B)** SWFD_sc, **(C)** SCD_ms, **(D)** SCD_vc, and **(E)** EFA.

Fig. 6 shows scatter plots of predicted versus reference scores for S3IM (columns 1 to 3) and SSIM (columns 4 to 6) across datasets and models. For the in-distribution datasets, SWFD_sc and SCD_ms, all three models cluster tightly along the diagonal, indicating high predictive accuracy and low error. On SSIM for SCD_ms (C4 to C6), PAQNet and IQDCNN exhibit slightly less dispersion than EfficientNetIQA, suggesting more stable predictions. On the Mice dataset, EfficientNetIQA shows less deviation compared to the true value (diagonal), than the other models for both SSIM and S3IM (A3, A6), consistent with its lower MAE in Table 5. In contrast, the EFA dataset shows the weakest correspondence for all models (E1 to E6), with broader spread and bias from the diagonal, matching the higher errors in Table 5. Across datasets, PAQNet tends to yield tighter SSIM distributions (right panels), while EfficientNetIQA is competitive for S3IM (left panels), indicating that predictive reliability depends on both dataset and metric.

**Table 6:** Computational efficiency of PAQNet, IQDCNN, and EfficientNetIQA for 128 × 128 grayscale inputs (batch=1). Operation counts are tool-estimated per forward pass; inference time measured on an NVIDIA GPU (CUDA).

Model	Parameters (M)	FLOPs (G)	Inference Time (ms)	Memory (MB)
**PAQNet**	**2.50**	0.246	**0.59 ± 0.12**	**19**
IQDCNN	3.78	0.152	0.64 ± 0.01	21
EfficientNetIQA	4.17	**0.126**	7.05 ± 0.22	73

The correlation results in Table 7 further support the observed performance trends. For the in-distribution datasets (SWFD_sc, SCD_ms, Mice), correlations between predicted and true scores are consistently high. In particular, SWFD_sc shows near-perfect agreement across all models, with Pearson r>0.97 and Spearman ρ>0.96. On the SCD_ms dataset, EfficientNetIQA achieves the highest correlations for SSIM (ρ=0.9584, r=0.9701), while PAQNet leads slightly on S3IM (ρ=0.6809, r=0.6981). Similarly, on the Mice dataset, PAQNet obtains the strongest correlations for both S3IM (ρ=0.9107, r=0.9460) and SSIM (ρ=0.9230, r=0.9653), highlighting its stability in this setting.

**Table 7:** Spearman and Pearson correlation between predicted and true S3IM and SSIM values across datasets.

Model	Mice	SWFD_sc	SCD_ms	SCD_vc	EFA
**Spearman (S3IM)**					
PAQNet	0.9107	0.9799	0.6809	0.8716	−0.0059
IQDCNN	0.7856	0.9648	0.5989	0.8445	0.8429
EfficientNetIQA	0.7686	0.9814	0.7742	0.8601	0.6608

**Pearson (S3IM)**					
PAQNet	0.9460	0.9808	0.6981	0.9262	0.0596
IQDCNN	0.8227	0.9766	0.6045	0.8898	0.8084
EfficientNetIQA	0.8784	0.9870	0.7541	0.8313	0.4490

**Spearman (SSIM)**					
PAQNet	0.9230	0.9746	0.9166	0.8851	0.8202
IQDCNN	0.8260	0.9672	0.9040	0.8654	0.6645
EfficientNetIQA	0.8258	0.9755	0.9584	0.8642	0.6865

**Pearson (SSIM)**					
PAQNet	0.9653	0.9811	0.9819	0.9304	0.6462
IQDCNN	0.8531	0.9751	0.9584	0.8820	0.6763
EfficientNetIQA	0.8871	0.9738	0.9701	0.9023	0.4633

In contrast, the EFA dataset shows markedly reduced correlations, reflecting the domain gap caused by its different imaging setup. Note that model prediction uncertainty remains higher for out-of-distribution data (EFA), confirming limits of domain generalization. For S3IM, IQDCNN generalizes best (ρ=0.8429, r=0.8084), while both PAQNet and EfficientNetIQA show weak or inconsistent correlations (ρ=−0.0059 and 0.6608 respectively). For SSIM, PAQNet achieves the highest correlations (ρ=0.8202, r=0.6462), whereas EfficientNetIQA lags behind (ρ=0.6865, r=0.4633).

All three models effectively reproduce full-reference metric scores for in-distribution PAI images, with EfficientNetIQA generally leading in SSIM correlations and competitive on S3IM. However, their ability to generalize to unseen domains such as EFA is limited, underscoring the need for more diverse training data or domain adaptation strategies.

**Fig. 7:**
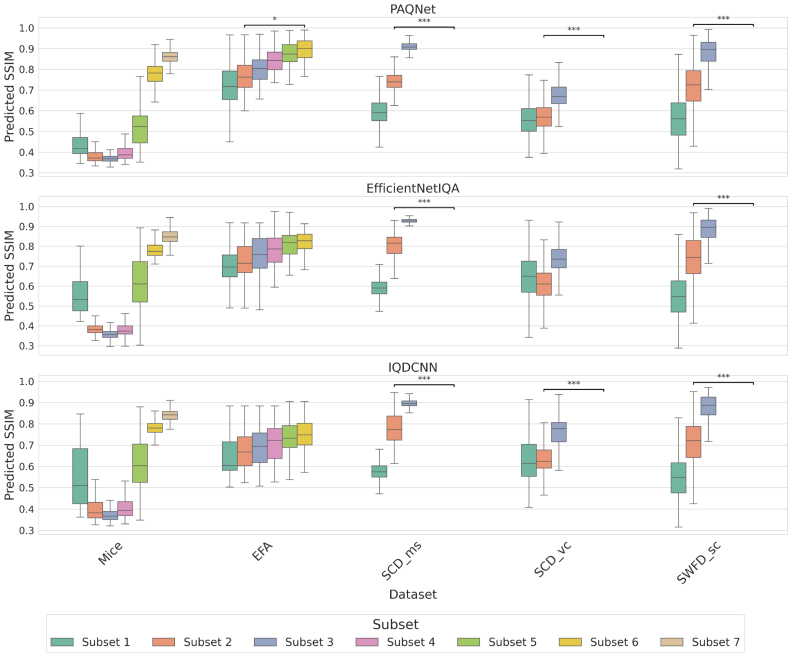
Predicted **SSIM** scores using PAQNet, EfficientNetIQA and IQDCNN across datasets, with subset labels explained in Table 2.

Fig. 7 shows predicted SSIM across datasets, grouped by subsets that reflect increasing image quality, consistent with the configuration order in Fig. 2, Fig. 3 and the dataset definitions in Section 4.1.1. PAQNet and IQDCNN recover the expected ordering with clear separation for *SCD_ms*, *SCD_vc*, and *SWFD_sc*, consistent with their in-distribution status relative to OADAT. For *SCD_vc*, EfficientNetIQA does not preserve the full subset order. On *Mice*, ordering is less consistent, with the first subset predicted higher than subsequent ones before increasing again, deviating from the full-reference trends in Fig. 2, Fig. 3. For *EFA*, the monotonic trend with increasing frame averaging is largely preserved, and PAQNet shows several significant differences between subsets. Notably, this occurs despite higher overall error on EFA, indicating that models can still track relative quality changes even when absolute prediction accuracy degrades. This behavior aligns with the correlation patterns in Table 7, which are strong in-distribution and reduced for EFA.

**Fig. 8:**
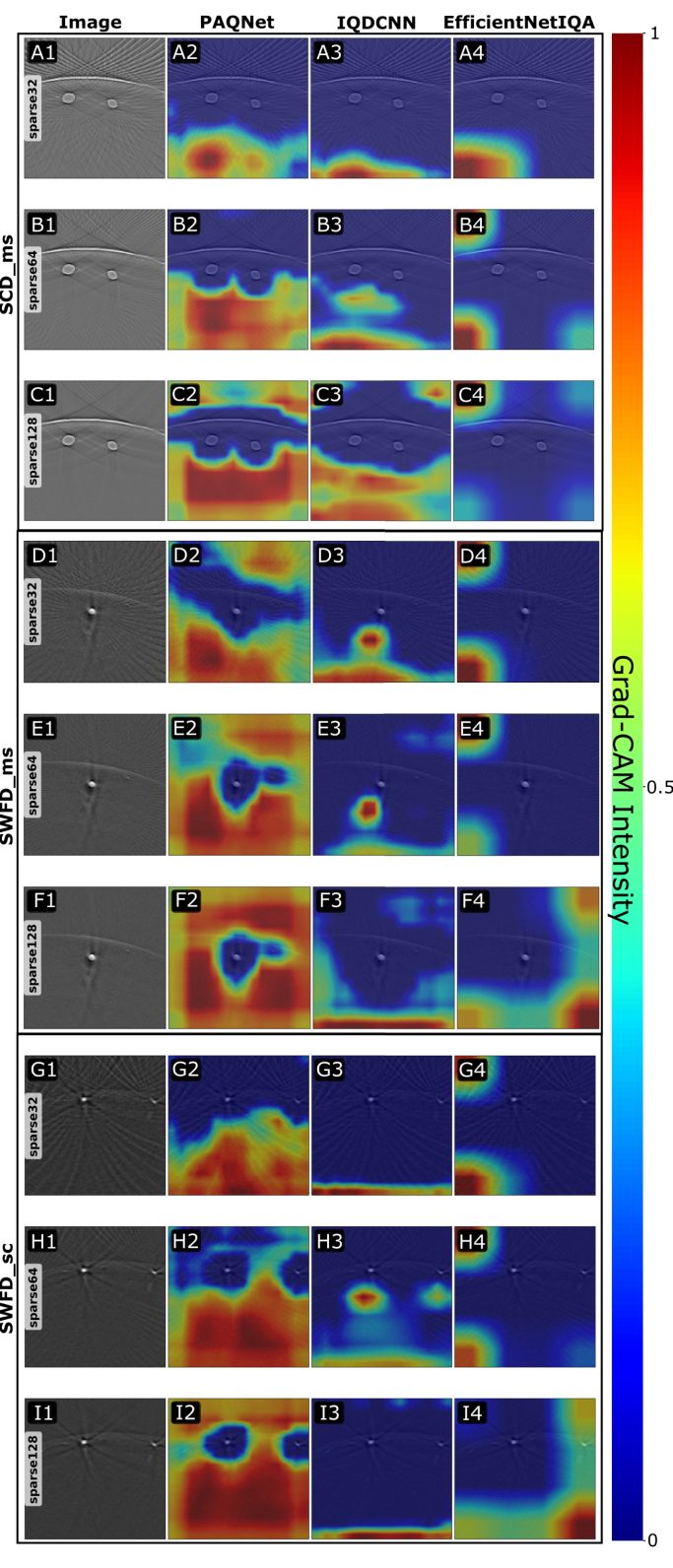
Grad-CAM intensity maps of images from three different datasets SWFD_sc, SWFD_ms, and SCD_ms with three different sparse transducers levels 32, 64 and 128. Red areas indicate strong positive Grad-CAM activations (intensity ≈1) while blue areas indicate weak activations (intensity ≈0). Each panel shows the original reconstruction and Grad-CAM overlays for PAQNet, IQDCNN, and EfficientNetIQA.

**Grad-CAM Analysis**: To examine the features influencing our quality prediction model, we generated Grad-CAM visualizations for reconstructions obtained with 32, 64, and 128 detectors for the SWFD_ms dataset, shown in Fig. 8. For the proposed model, PAQNet, the maps mainly highlighted noise-dominated regions. In the 32 detector reconstructions, where structural detail is limited, they emphasized areas with reduced signal consistency. For the 64 and 128 detector cases, where anatomical structures are better recovered, the attention remained on background noise and showed low response to anatomy. This indicates that the model bases its predictions on noise-related degradations and is also sensitive to streaking artefacts. In contrast, the attention maps for IQDCNN and EfficientNetIQA rely on restricted regions, often at the corners or lower parts of the image, which makes their quality estimates less reliable.

### 4.3 Ablation studies: Multi-metric training and model interpretability

**Multi-metric Training:** As shown in Table 8, multi-metric training does not consistently lead to lower MAE (L1) losses compared to training on single metrics. Here, we define Δ= (MAE of combination) − (mean MAE of corresponding single metrics). Thus, negative Δ values indicate that the combined training performed better than the average of the individual metrics. The results in Table 9 show that PAQNet benefits slightly from all three two-metric pairings (−0.0055, −0.0058, −0.0066), while EfficientNetIQA improves for SSIM+GMSD (−0.0066) and SSIM+HAARPSI (−0.0011).

In contrast, IQDCNN shows small gains only for SSIM+HAARPSI (−0.0008) and performs worse when all five metrics are combined (+0.0336). Overall, targeted pairings can provide modest benefits, but combining many metrics indiscriminately offers little advantage and can even harm performance.

**Table 8:** MAE loss per dataset and metric combination (Grouped by model).

Dataset	Model	SSIM GMSD	SSIM HAARPSI	GMSD HAARPSI	SSIM GMSD HAARPSI S3IM IW-SSIM
Mice	PAQNet	0.1623	**0.1431**	**0.2313**	**0.1606**
	IQDCNN	0.1516	0.1766	0.2458	0.1805
	EfficientNetIQA	**0.1260**	0.1525	0.2508	0.2135

SWFD_sc	PAQNet	0.0321	0.0301	0.0368	0.0307
	IQDCNN	0.0336	0.0381	0.0374	0.0442
	EfficientNetIQA	**0.0213**	**0.0206**	**0.0286**	**0.0257**

SCD_vc	PAQNet	**0.1186**	0.1399	**0.1674**	0.1299
	IQDCNN	0.1465	0.1465	0.1806	0.1487
	EfficientNetIQA	0.1281	**0.1338**	0.1913	**0.1165**

EFA	PAQNet	0.4582	0.4493	0.4848	0.5085
	IQDCNN	0.3983	**0.3652**	**0.3585**	0.4831
	EfficientNetIQA	**0.3636**	0.4087	0.4023	**0.4722**

SCD_ms	PAQNet	0.0426	**0.0313**	**0.0399**	**0.0325**
	IQDCNN	0.0492	0.0325	0.0545	0.0559
	EfficientNetIQA	**0.0417**	0.0388	0.0583	0.0447

Average	PAQNet	0.1622	0.1580	0.1913	**0.1728**
	IQDCNN	0.1556	0.1518	**0.1755**	0.1821
	EfficientNetIQA	**0.1363**	**0.1507**	0.2067	0.1749

**Table 9:** Average difference (Δ) in MAE loss between multi-metric training and the mean of the corresponding single-metric trainings. A negative Δ indicates that the combination achieves lower (better) loss.

Model	SSIM+GMSD	SSIM+HAARPSI	GMSD+HAARPSI	All Five
PAQNet	−0.0055	−0.0058	−0.0066	0.0004
IQDCNN	0.0016	−0.0008	0.0061	0.0336
EfficientNetIQA	−0.0066	−0.0011	0.0557	0.0243

## 5 Discussion

This work represents, to our knowledge, the first systematic study of image quality assessment (IQA) in photoacoustic imaging (PAI). By benchmarking a wide set of metrics and introducing learning-based models, we establish which approaches are most effective and where major challenges remain.

### 5.1 Implications for PAI quality assessment

Our findings show that metrics emphasizing structural similarity, such as SSIM and its variants, are the most reliable for PAI. This reflects the importance of preserving vascular and edge detail in reconstructions. In contrast, intensity-based measures like PSNR do not capture clinically relevant differences, for example distinguishing blur from noise. For objective reporting and comparison of reconstructions, metrics such as SSIM, S3IM, IW-SSIM, GMSD or HAARPSI are therefore the most suitable.

The ROI-based analysis confirms that increasing the number of receive channels improves target detectability in practice. SNR${}_{\mathrm{dB}}$ and gCNR rose consistently in all datasets, with the largest gains in SWFD_sc, indicating clearer separation of targets from their local background. The small and sometimes negligible changes in CNR are expected because CNR scales with the background variance and is less sensitive when both signal and background fluctuate together. In contrast, gCNR reflects distribution overlap, so it tracks detectability more faithfully and aligns with the improvements seen in our full-image metrics. These findings show that the ROI-based metrics can be used as no-reference metric. The primary limitation of these metrics is that manual region selection is required in this case. Hence, these metrics can be used when automated region selection is possible. We found that SNR and gCNR are more effective in highlighting the quality differences. These metrics can be used as no-reference metrics when the targets are well-known and can be automatically selected.

Generic no-reference (NR) metrics from natural imaging, such as BRISQUE or CLIP-IQA, performed poorly. Their assumptions about natural scene statistics do not hold in PAI, where background noise, wavelength dependence, and depth-related attenuation dominate. This underscores the need for NR metrics tailored to PAI characteristics and artefacts.

Learning-based NR models, particularly EfficientNetIQA, successfully predicted FR metric scores on in-distribution data, enabling reference-free quality assessment. Such models could provide real-time quality feedback during acquisition. Effective deployment will require training on broader datasets or adopting domain adaptation methods. Although the models do not predict the exact absolute metric values, they effectively mimic the trend of full-reference (FR) scores, consistent with their intended use as no-reference (NR) estimators. For in-distribution datasets such as *Mice*, *SWFD*, and *SCD*, PAQNet achieves low mean absolute errors (e.g., MAE${}_{\text{SSIM}}$ = 0.083–0.038) and high correlation with true scores (Spearman ≥ 0.88, Pearson ≥ 0.93), demonstrating strong alignment between predicted and actual metric trends. The scatter plots in Fig. 6 further confirm this correspondence, showing tight clustering along the diagonal for in-distribution data. Only the *EFA* dataset exhibits larger deviations, which is expected given its distinct imaging conditions and unseen domain characteristics. This highlights the current limitation in domain generalization and supports the need for explicit domain adaptation.

Interpretability analysis showed that models often attend to meaningful structures but occasionally misinterpret artefacts as quality features. This mismatch indicates that high numerical correlation with FR metrics does not guarantee clinically aligned judgments. Improving interpretability should be a priority to ensure safe application in medical contexts.

### 5.2 Limitations and future directions

Our models were trained on FR metrics, which themselves are proxies for human perception and diagnostic utility. While necessary in the absence of large-scale subjective ratings, this constrains what the models can learn. The datasets, although extensive, lack clinical patient images and therefore may not cover the variability present in real clinical practice. Finally, our exploration of multi-metric training was limited to a few combinations, leaving room for more systematic investigation.

A key limitation of our ranking framework is the absence of an external ground truth based on human observers or diagnostic tasks. We implicitly treat the acquisition induced quality levels as reference categories and evaluate how consistently each metric separates them. Although this approach enables systematic, large scale comparison across multiple datasets and artefact types, it does not replace subjective quality assessment with human subjects. Future work should therefore complement the present analysis with expert studies and application specific performance metrics.

Future work should prioritize the development of PAI-specific NR metrics, either through analytic approaches informed by domain knowledge or through models trained on human preference and task-performance data. Integrating IQA into reconstruction algorithms is another promising direction, allowing systems to optimize parameters based on predicted quality during acquisition. To improve generalization, domain adaptation and multi-domain training strategies should be pursued. Enhancing interpretability through architectures designed for transparent decision-making will also be important for clinical acceptance. Finally, since IQA scores vary across datasets from different systems, combining multiple metrics into a single learning-based composite score may provide a more robust and transferable assessment.

### 5.3 Broader impact

Reliable IQA will be central to advancing PAI towards clinical translation. Embedding automated quality assessment into imaging systems would allow non-technical users to monitor and optimize acquisitions in real time. Standardized, objective quality measures will also accelerate fair comparison of algorithms and hardware. Automated and interpretable quality prediction can support clinical adoption by detecting low-quality acquisitions early and by quantifying improvements. More broadly, this study shows that although no single metric captures all aspects of image quality, carefully designed machine learning approaches can provide practical and generalizable solutions when trained on diverse data.

## 6 Conclusion

We presented a comprehensive evaluation of image quality assessment (IQA) methods for photoacoustic imaging, combining traditional full-reference metrics with recent learning-based models. Using nearly one million PA images from diverse datasets, we benchmarked eleven full-reference and two no-reference metrics, and trained three deep neural networks to perform no-reference quality prediction by mimicking full-reference measures. The results show that structural similarity metrics such as SSIM, S3IM, and IW-SSIM were the most reliable in capturing reconstruction quality, surpass intensity-based metrics like PSNR and generic image statistics. In contrast, general-purpose no-reference metrics such as BRISQUE and CLIP-IQA correlated poorly with reconstruction quality, highlighting the need for PAI-specific or learned approaches. Our deep learning models, particularly EfficientNetIQA, achieved strong performance with correlations up to 0.95 against reference-based scores, providing a practical full-reference proxy without requiring reference images. However, performance decreased when models were applied across different data distributions, such as across different PAI systems, indicating that generalization remains a challenge and will require broader training strategies or domain adaptation. Finally, while combining multiple metrics within a single training framework yielded marginal improvements in isolated cases, it did not consistently enhance prediction accuracy and sometimes degraded it, suggesting that selective integration of complementary metrics is preferable. This study establishes a foundation for both benchmarking and advancing no-reference IQA methods tailored to PAI, with deep learning models offering a promising path towards reference-free quality assessment in practical applications.

## CRediT authorship contribution statement

**Melle Van Der Brugge:** Writing – original draft, Visualization, Validation, Software, Methodology, Investigation, Formal analysis, Data curation. **Kalloor Joseph Francis:** Writing – review & editing, Validation, Supervision, Resources, Project administration, Methodology, Investigation, Conceptualization. **Navchetan Awasthi:** Writing – review & editing, Validation, Supervision, Resources, Project administration, Methodology, Investigation, Conceptualization.

## Funding

This work has received financial support from the Dutch Research Council (NWO) for the project NWO-VENI (19165), 10.13039/501100001826ZonMw for the project Off Road (04510012210042).

## Declaration of competing interest

The authors declare that they have no known competing financial interests or personal relationships that could have appeared to influence the work reported in this paper.

## Data Availability

We have shared the code repository for all the codes used in this work at https://github.com/MellevdB/photoacoustic-image-quality-assessment.git.

## Appendix A Supplementary data

MMC S1 . MMC S1
