# Supplementary material for: Assessing image quality in photoacoustic imaging: A metric-based and deep learning-based evaluation
Source: Photoacoustics. 2026 Feb 28;49:100800. doi: 10.1016/j.pacs.2026.100800 (PMC12991846; doi:10.1016/j.pacs.2026.100800)
Supplement: MMC S1 — . [file mmc1.pdf]

Supplementary Material: Assessing Image Quality in Photoacoustic Imaging: A Metric-Based and Deep Learning-Based Evaluation

Melle Van Der Brugge[1], Kalloor Joseph Francis[2], Navchetan Awasthi[1,3]

[1] Faculty of Science, Mathematics and Computer Science, Informatics Institute, University of Amsterdam, Amsterdam, The Netherlands
[2] Erasmus MC, Cardiovascular Institute, Department of Cardiology, Biomedical Engineering, Rotterdam, The Netherlands
[3] School of Artificial Intelligence and Data Science, IIT Jodhpur, India

1 Example Images

Here, we provide representative example images for each dataset used in this study, in order to give visual context independent of the main manuscript. The datasets include MSFD, SCD (multisegment), SCD (virtual circle), SWFD (multisegment), SWFD (semi circle / single-segment), Mice (in vivo), Experimental Phantom, Virtual Phantom, Experimental Frame Averaging (EFA; KneeSlice, Breast Phantom, SmallAnimal, Transducers), and Neurovascular Network Explorer (NNE). These examples illustrate the diversity in configurations, reconstruction, and imaging targets, ranging from simulated phantoms to in vivo measurements. The images show differences, artifact patterns, resolution characteristics, and anatomical or structural content, which form the basis for the quantitative image quality analyses presented in the study.

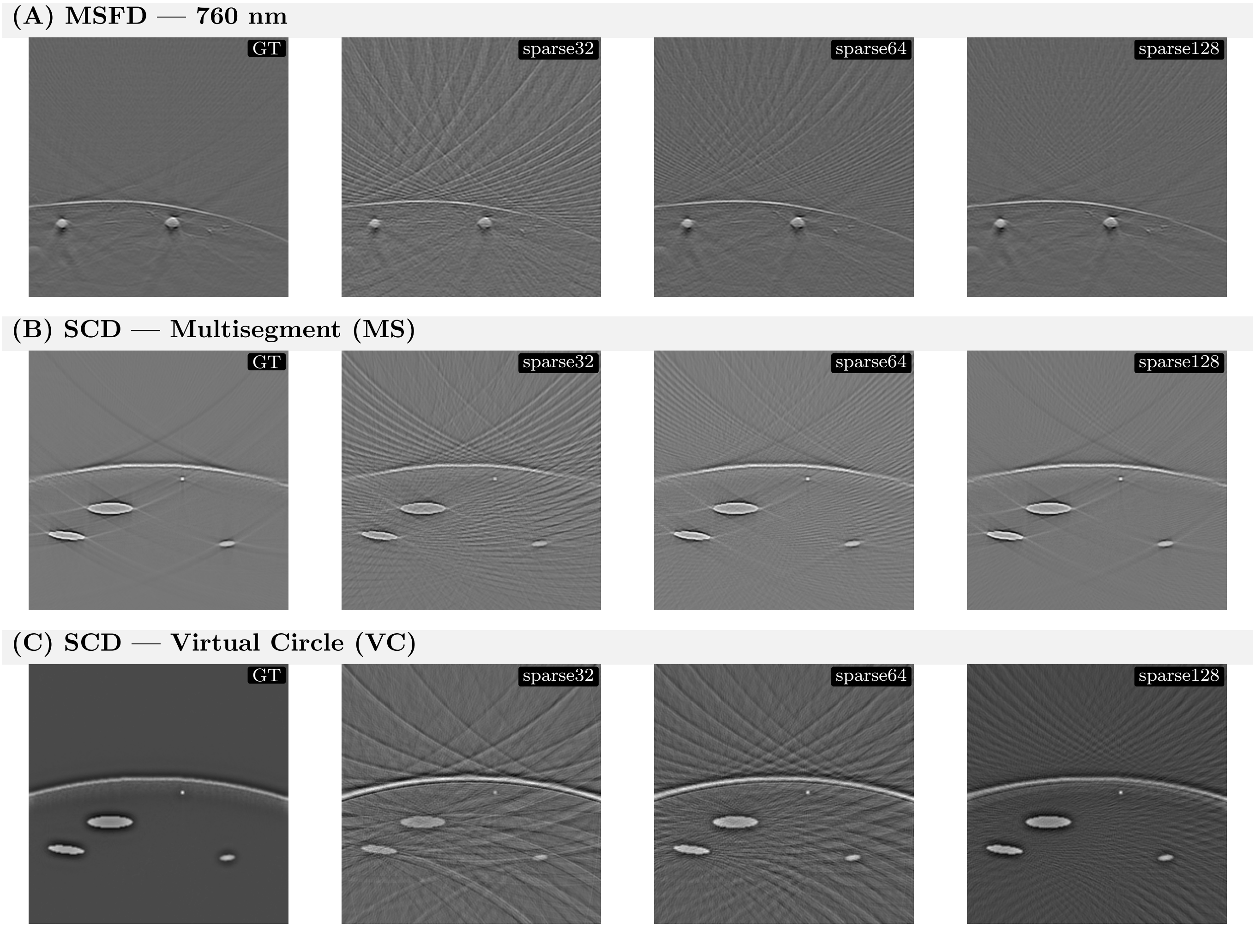

Figure 1: OADAT examples (Part 1). GT followed by progressively sparser configurations.

(D) SWFD — Multisegment (MS)

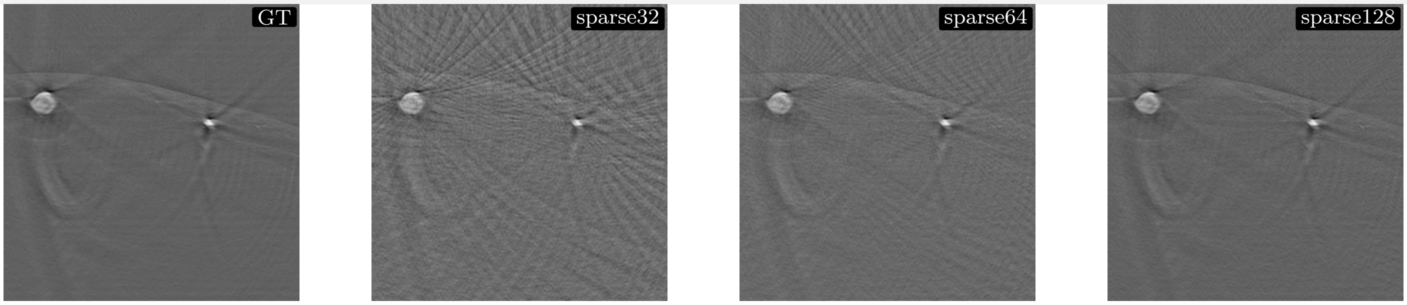

(E) SWFD — Semicircle / Single-segment (SC)

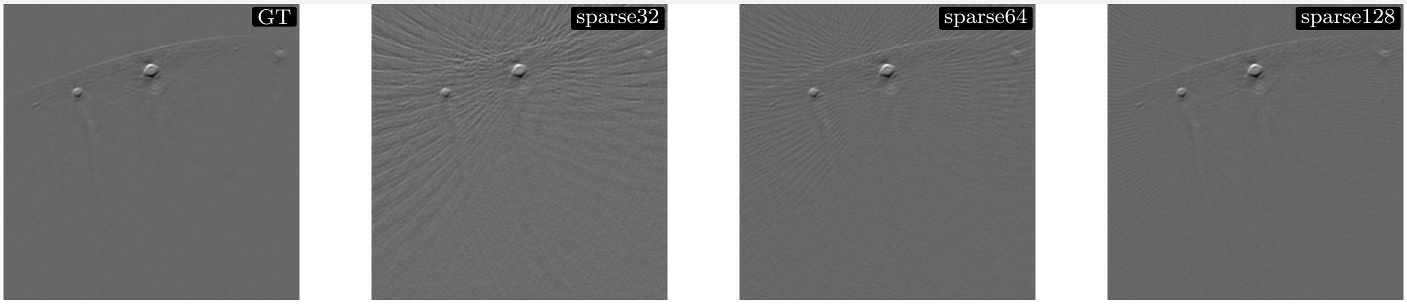

(F) Mice (*in vivo*) — Sparse sampling

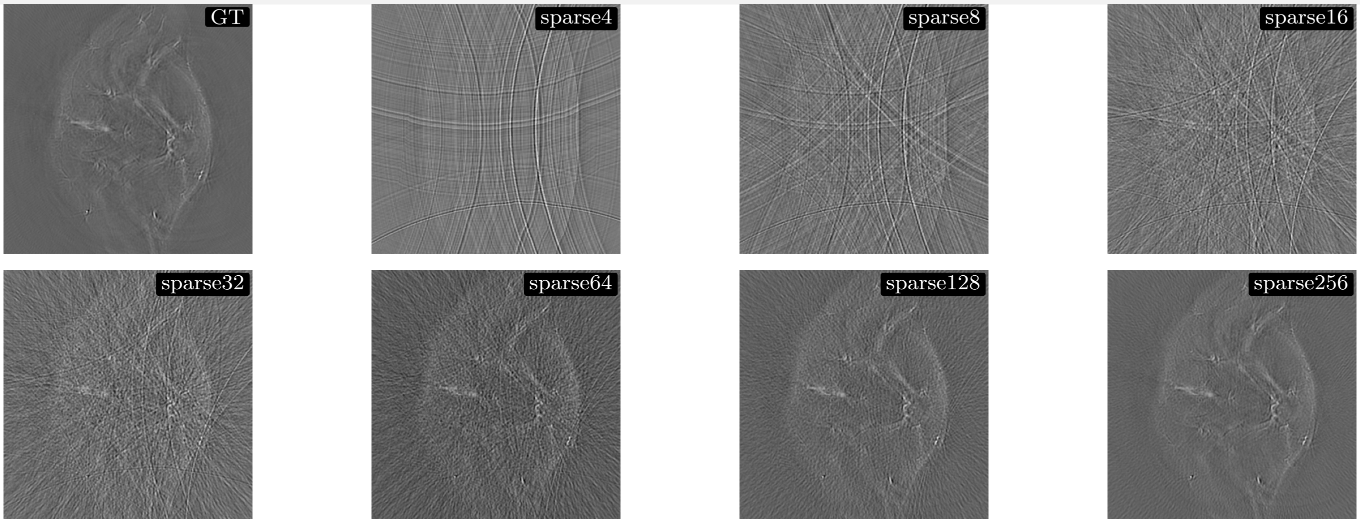

(G) Experimental Phantom — Sparse sampling

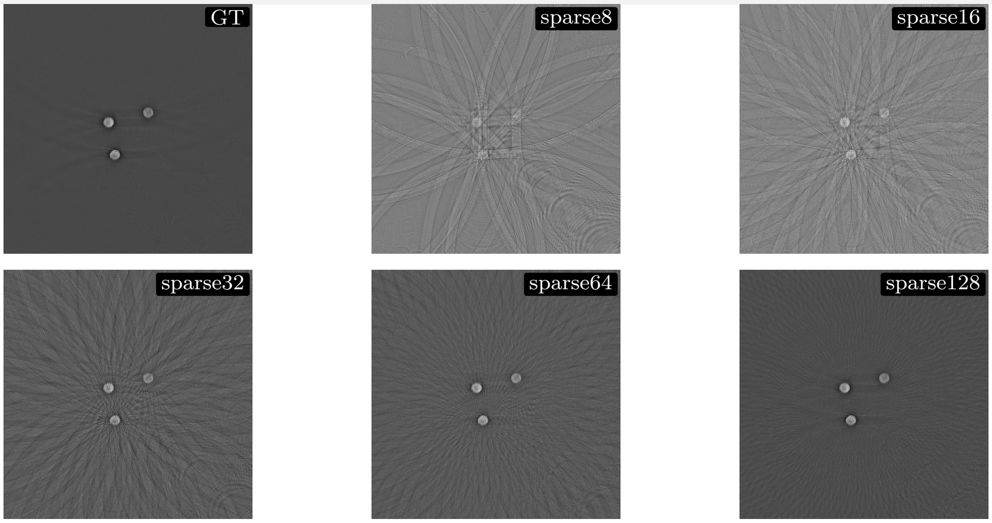

Figure 2: OADAT SWFD examples, followed by full mice and experimental phantom grids.

(H) Virtual Phantom (simulated) — Sparse sampling

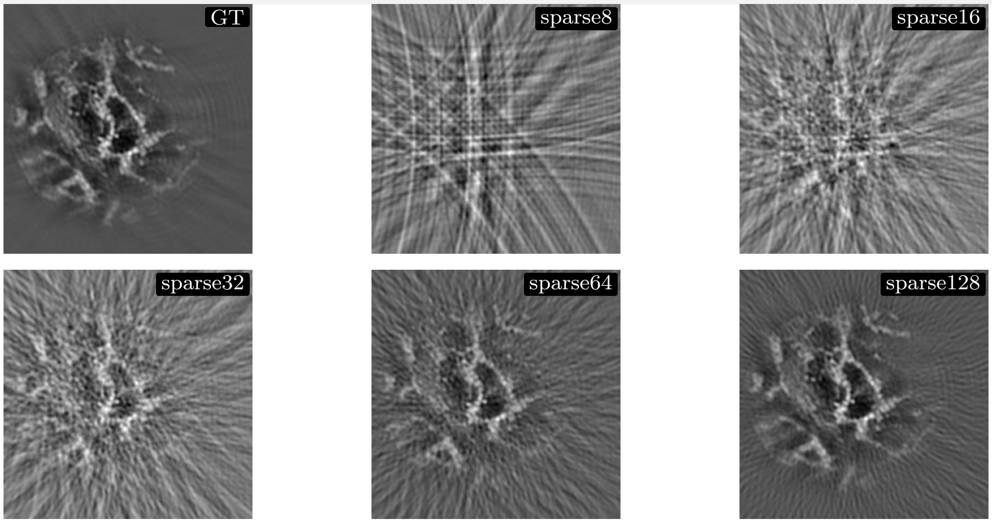

(I) Experimental Frame Averaging (EFA) — KneeSlice

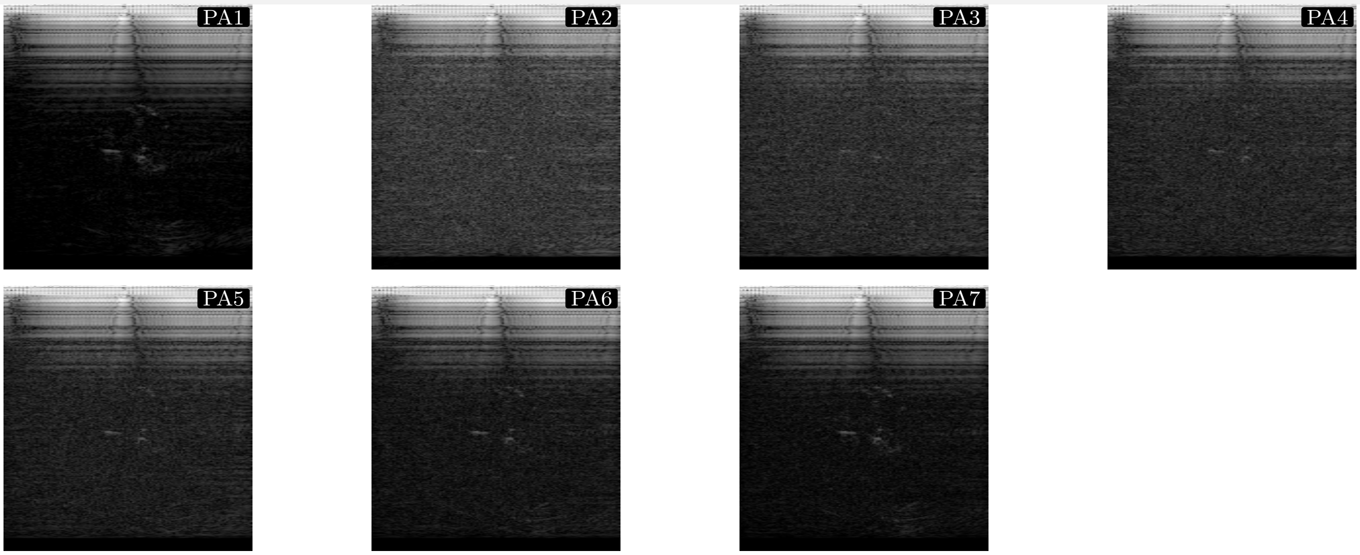

(J) EFA — Breast Phantom

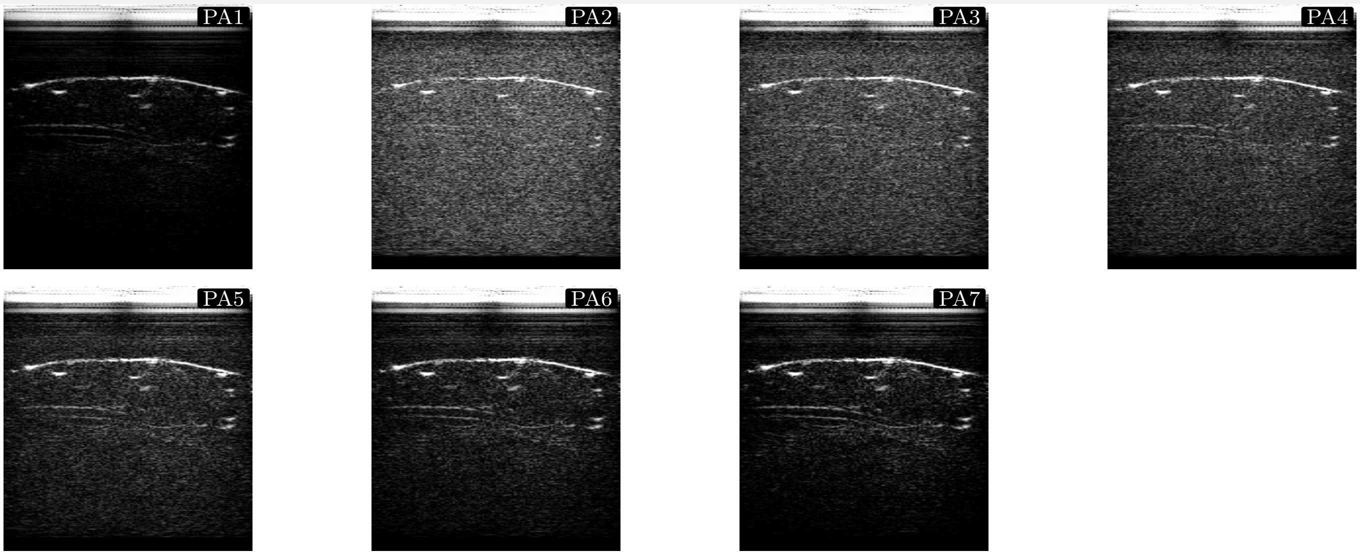

Figure 3: Virtual Phantom grid, followed by EFA subsets: KneeSlice and Breast Phantom with all frame-averaging levels (PA1–PA7).

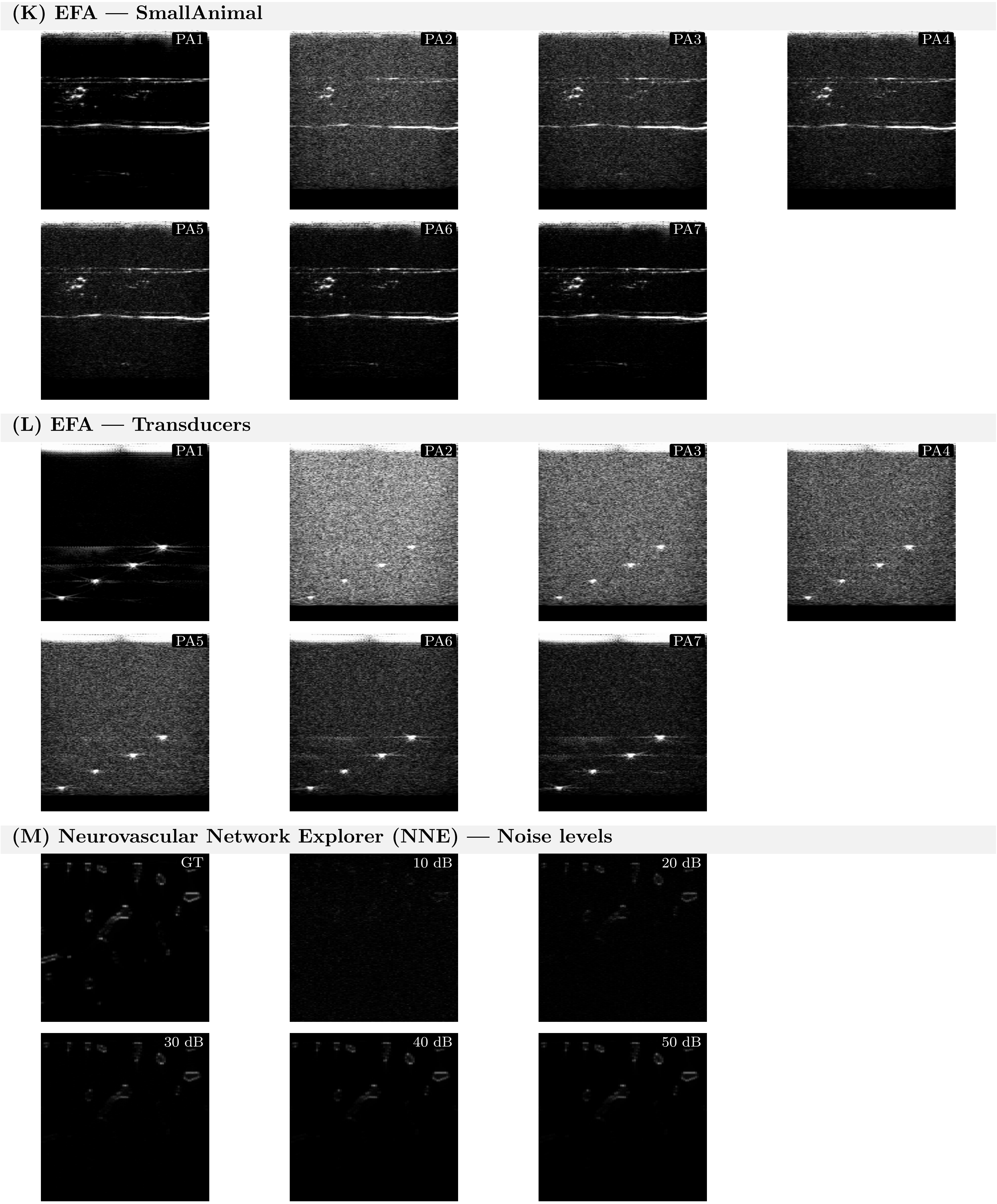

Figure 4: EFA SmallAnimal and Transducers with all levels (PA1–PA7), and NNE with all noise settings (GT, 10–50 dB).

1.1 Supplementary Error/Confidence Analysis (Mean ± Std)

This appendix complements the main comparisons in Figures 2 and 3.

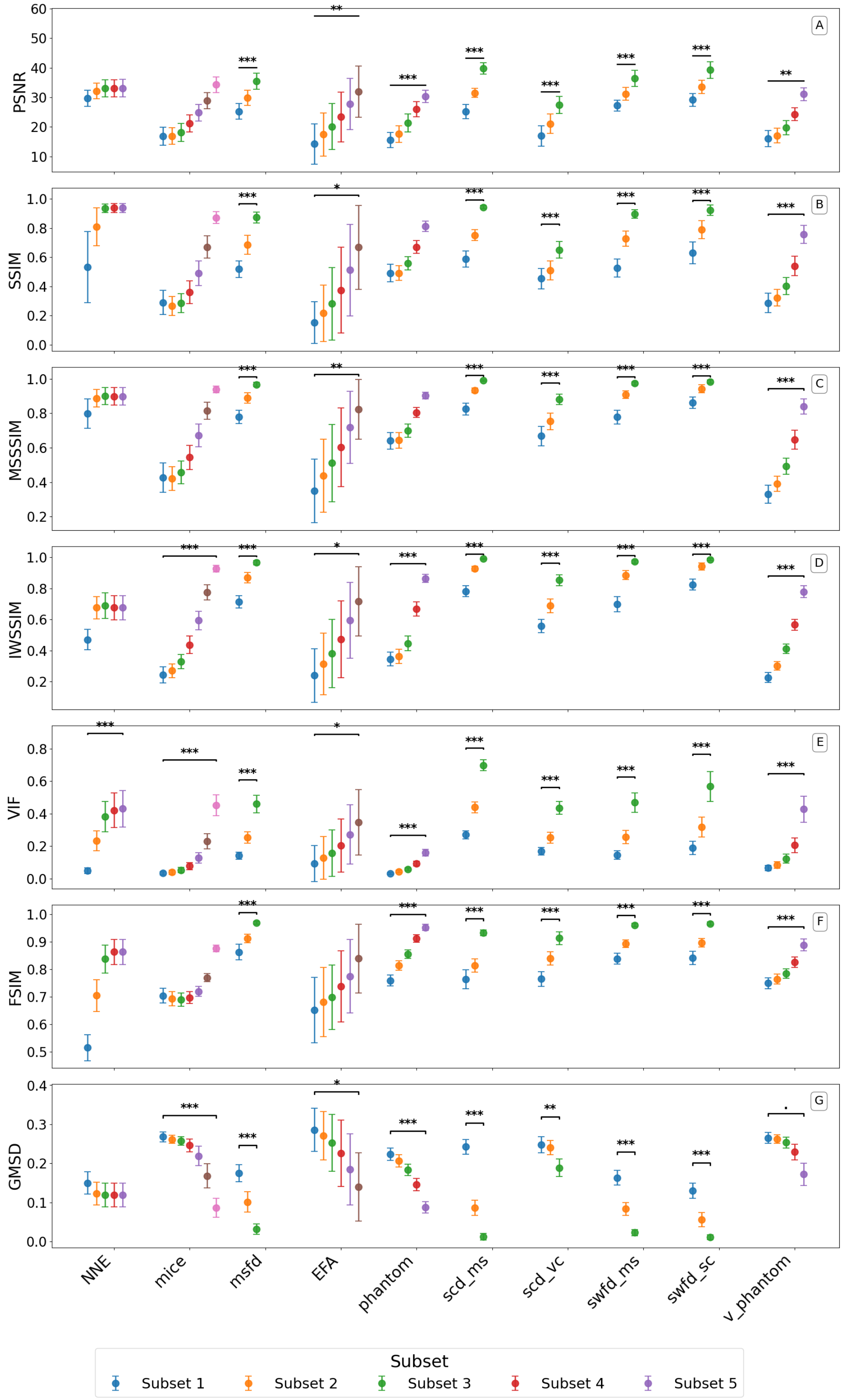

Figure 5: Tukey-style boxplotof metric distributions (page 1 of 2) on various datasets with subsets representing different configurations or settings resulting in different quality levels. Mean ± std per metric and configuration. This figure complements Figure 2 in the article.

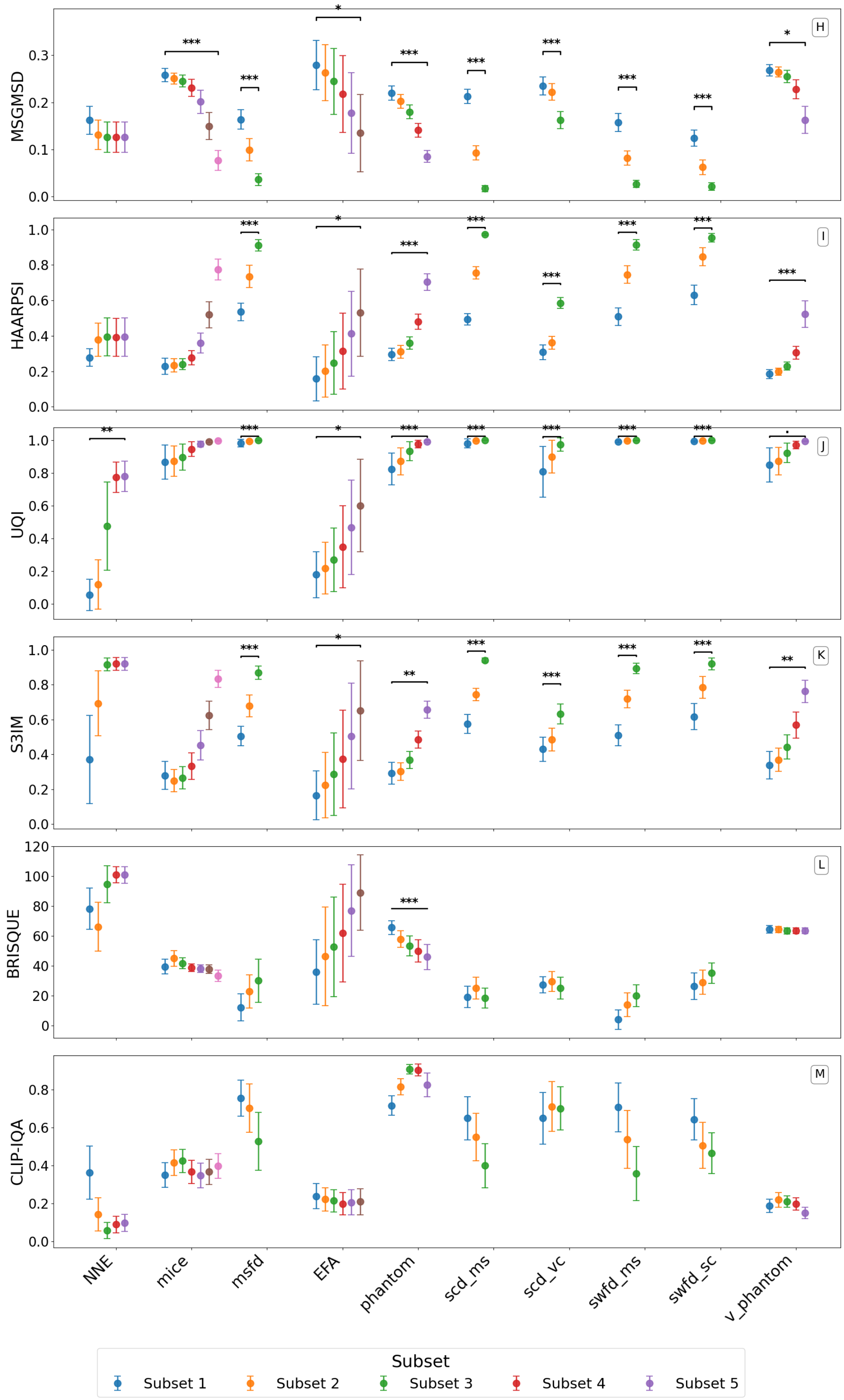

Figure 6: Tukey-style boxplot of metric distributions (page 2 of 2) on various datasets with subsets representing different configurations or settings resulting in different quality levels. Mean ± std per metric and configuration. This figure complements Figure 3 in the article.

In addition to the Tukey-style boxplots(showing median, interquartile range, whiskers up to 1.5×IQR, and outliers) shown in the main manuscript, these two figures presents complementary boxplots summarizing the mean metric values per subset within each dataset, together with their standard deviations.
